# *Lactobacillus plantarum* Disrupts *S. mutans–C. albicans* Cross-Kingdom Biofilms

**DOI:** 10.3389/fcimb.2022.872012

**Published:** 2022-03-22

**Authors:** Yan Zeng, Ahmed Fadaak, Nora Alomeir, Tong Tong Wu, Elena Rustchenko, Shuang Qing, Jianhang Bao, Christie Gilbert, Jin Xiao

**Affiliations:** ^1^ Eastman Institute for Oral Health, University of Rochester Medical Center, Rochester, NY, United States; ^2^ Department of Biostatistics and Computational Biology, University of Rochester Medical Center, Rochester, NY, United States; ^3^ Department of Biochemistry and Biophysics, University of Rochester Medical Center, Rochester, NY, United States; ^4^ University of Rochester River Campus, Rochester, NY, United States; ^5^ Microbiology and Immunology, University of Rochester Medical Center, Rochester, NY, United States

**Keywords:** *Streptococcus mutans*, *Candida albicans*, dental caries, multispecies biofilms, cross-kingdom interactions, *Lactobacillus plantarum*

## Abstract

Dental caries, an ecological dysbiosis of oral microflora, initiates from the virulent biofilms formed on tooth surfaces where cariogenic microorganisms metabolize dietary carbohydrates, producing acid that demineralizes tooth enamel. Forming cariogenic biofilms, *Streptococcus mutans* and *Candida albicans* are well-recognized and emerging pathogens for dental caries. Recently, probiotics have demonstrated their potential in treating biofilm-related diseases, including caries. However, limited studies have assessed their effect on cariogenic bacteria–fungi cross-kingdom biofilm formation and their underlying interactions. Here, we assessed the effect of four probiotic *Lactobacillus strains (Lactobacillus rhamnosus* ATCC 2836, *Lactobacillus plantarum* ATCC 8014, *Lactobacillus plantarum* ATCC 14917, and *Lactobacillus salivarius* ATCC 11741) on *S. mutans* and *C. albicans* using a comprehensive multispecies biofilm model that mimicked high caries risk clinical conditions. Among the tested probiotic species, *L. plantarum* demonstrated superior inhibition on the growth of *C. albicans* and *S. mutans*, disruption of virulent biofilm formation with reduced bacteria and exopolysaccharide (EPS) components, and formation of virulent microcolonies structures. Transcriptome analysis (RNA sequencing) further revealed disruption of *S. mutans* and *C. albicans* cross-kingdom interactions with added *L. plantarum*. Genes of *S. mutans* and *C. albicans* involved in metabolic pathways (e.g., EPS formation, carbohydrate metabolism, glycan biosynthesis, and metabolism) were significantly downregulated. More significantly, genes related to *C. albicans* resistance to antifungal medication (ERG4), fungal cell wall chitin remodeling (CHT2), and resistance to oxidative stress (CAT1) were also significantly downregulated. In contrast, *Lactobacillus* genes *plnD*, *plnG*, and *plnN* that contribute to antimicrobial peptide plantaricin production were significantly upregulated. Our novel study findings support further assessment of the potential role of probiotic *L. plantarum* for cariogenic biofilm control.

## 1 Introduction

Dental caries, an ecological dysbiosis of oral microflora, initiates from the virulent biofilms formed on tooth surfaces where cariogenic bacteria and fungi metabolize dietary carbohydrates, produce acid, and lead to irreversible consequences—demineralization of tooth enamel (Bowen, 2016). *Streptococcus mutans* is a well-known cariogenic pathogen due to its acidogenicity, aciduricity, and capability of synthesizing the dental plaque extracellular matrix (Bowen et al., 2018). Moreover, research also revealed the cariogenic role of oral *Candida*, in that it is acidogenic, aciduric, and capable of dissolving hydroxyapatite and leads to more severe dental caries when infected together with *S. mutans* in the rat model (Falsetta et al., 2014; Du et al., 2021). Children with oral *Candida albicans* presented with >5 times greater odds of experiencing early childhood caries (ECC) than children without this yeast strain (Xiao et al., 2018b). The presence of *C. albicans* in the oral cavity of preschool children was associated with oral bacterial dysbiosis with an abundance of taxa with greater virulence and more conducive for ECC (Xiao et al., 2018a). Furthermore, the emergence of *S. mutans* by 1 year was 3.5 times higher in infants with early colonization of oral *Candida* than those free of oral *Candid*a (Alkhars N et al., 2021). Therefore, regulating *S. mutans* and *C. albicans* simultaneously in the oral cavity sheds new light on caries prevention.

Probiotic therapy has the potential to prevent and treat dental caries (Zaura and Twetman, 2019). The most commonly used probiotics include *Lactobacilli* and *Bifidobacterium*, both of which produce lactic acid and other bioactive substances, including hydrogen peroxide, carbon peroxide, bacteriocins, and adhesion inhibitors that could potentially affect the growth of cariogenic microorganisms (Meurman, 2005). Several studies have reported the inhibitory effect of probiotics on *S. mutans* and *C. albicans*; for instance, *Lactobacillus rhamnosus*, *Lactobacillus reuteri*, *Lactobacillus casei*, *Lactobacillus plantarum*, and *Lactobacillus salivarius* inhibit the growth of *S. mutans in vitro* and *in vivo* (Laleman et al., 2014; Wasfi et al., 2018; Zhang et al., 2020a); *L. rhamnosus*, *L. casei*, *Lactobacillus paracasei*, *Lactobacillus fermentum*, and *Lactobacillus acidophilus* inhibit *C. albicans* biofilms (Matsubara et al., 2016; Rossoni et al., 2018; Panariello et al., 2021).

Since the coexistence of *S. mutans* and *C. albicans* in the oral cavity leads to a more pathogenic microbial eco-community and potentially elevates the caries risk of individuals (Koo et al., 2018; Du et al., 2021), an ideal probiotic regimen is to control *S. mutans* and *C. albicans* simultaneously. Intriguingly, our previous study revealed that the abundance of *L. plantarum* in dental plaques was three-fold higher in children without *C. albicans* compared to those with carriage of *C. albicans*, indicating a potential antagonistic interaction between *L. plantarum* and *C. albicans* (Xiao et al., 2018a). Moreover, Srivastava et al. reported that the supernatant of *L. plantarum* 108 inhibited the duo-species biofilm formation by *S. mutans* and *C. albicans* (Srivastava et al., 2020). Zhang et al. demonstrated that *L. plantarum* CCFM8724 could decrease the carriage of *S. mutans* and *C. albicans* in rat’s oral cavity and reduce caries score in rats (Zhang et al., 2020b). The abovementioned study findings support a better understanding of the interactions between *L. plantarum*, *S. mutans*, and *C. albicans* in multispecies biofilms. In the present study, we evaluated the effect of four probiotic *Lactobacillus strains* (two *L. plantarum*, *L. salivarius*, and *L. rhamnosus*) on the growth of *S. mutans* and *C. albicans*, in planktonic and cariogenic biofilm settings that simulate high caries risk clinical conditions. We used a comprehensive biofilm evaluation model and RNA-Seq analysis to reveal species interactions.

## 2 Materials and Methods

### 2.1 Bacterial Strains and Starter Preparation

The microorganisms used in the study were *S. mutans* UA159, *C. albicans* SC5314, *L. rhamnosus* ATCC 2836, *L. plantarum* ATCC 8014, *L. plantarum* ATCC 14917, and *L. salivarius* ATCC 11741. C*. albicans*, *S. mutans*, and *Lactobacillus* were recovered from frozen stock using YPD agar (BD Difco™, San Jose, CA, USA, 242720), blood agar (TSA with sheep blood, Thermo Scientific™, Waltham, MA, USA, R01202), and MRS agar (BD Difco™, 288210), respectively. After 48 h of incubation, 3–5 colonies of each species were inoculated into 10 ml of broth for overnight incubation (5% CO_2_, 37°C). *C. albicans* was grown in YPD broth (BD Difco™, 242820); *S. mutans* was grown in TSBYE broth (3% Tryptic Soy, 0.5% Yeast Extract Broth, BD Bacto™ 286220 and Gibco™ 212750) with 1% glucose; and *Lactobacillus* spp. were grown in MRS broth (BD Difco™, 288130). On the following day, 0.5 ml of the overnight starters was added to individual glass tubes with fresh broth and incubated for 3–4 h to reach the mid-exponential phase with desirable optical density. The morning starters were then ready for the preparation of planktonic and biofilm models described below.

### 2.2 Planktonic Model

Interactions between *C. albicans*, *S. mutans*, and *Lactobacillus* species were first evaluated in planktonic conditions; see **Figure S1A** for the study flow. The inoculation quantity of *C. albicans* (10^3^ CFU/ml) and *S. mutans* (10^5^ CFU/ml) was chosen to simulate high caries risk conditions in the clinical setting. The inoculation quantity of the four *Lactobacillus* (10^8^ CFU/ml) is the lower dose of the probiotics used in the commercial probiotic products (10^9^–10^12^ CFU as a single dosage). *C. albicans*, *S. mutans*, and one of the *Lactobacilli* were grown in 10 ml TSBYE broth with 1% glucose for 20 h (5% CO_2_, 37°C). Additionally, a dose-titration effect of *L. plantarum* 14917 (10^4^–10^7^ CFU/ml inoculation) was assessed. The growth of each microorganism and pH values were measured at multiple time points.

### 2.3 Mixed-Species Biofilm Model

We then used a mixed-species biofilm model to assess the effect of *Lactobacilli* on the biofilm formation by *S. mutans* and *C. albicans*; see **Figure S1B** for the study flow. The biofilm was formed on saliva-coated hydroxyapatite discs (0.50″ diameter × 0.05″ thickness, Clarkson Chromatography Products, Inc., South Williamsport, PA), the method detailed previously (Xiao et al., 2012). The discs were placed in a vertical position using a custom-made disc holder to mimic the caries-prone smooth tooth surfaces in the oral cavity (Xiao et al., 2012).

The mixture of *S. mutans*, *C. albicans*, and *Lactobacilli* was inoculated in 2.8 ml of TSBYE broth with 0.1% (w/v) sucrose and incubated at 37°C and 5% CO_2_. During the first 24 h, the organisms were grown undisturbed to allow initial biofilm formation. At 24 h, the biofilms were transferred to a fresh culture medium containing 1% (w/v) sucrose or 1% (w/v) glucose to induce cariogenic challenges, while an additional set of biofilms was grown with 0.1% sucrose. The culture medium was replaced every 24 h until the end of the experimental period (72 h). *Lactobacilli* (10^8^ CFU/ml) was added to the fresh culture medium daily. The culture medium pH was measured at selected time points. The biofilms underwent microbiological, dry-weight, and confocal imaging assays at 24, 48, and 72 h, transcriptome analysis *via* RNA-Seq at 48 h, and qRT-PCR validation at 48, 50, and 52 h. The methods are detailed previously (Xiao et al., 2012) and are shown in **Appendix 1**. Duplicated discs were used in each run. Independent assays were repeated three times.

### 2.4 Inhibition of *C. albicans* and *S. mutans* by *L. plantarum* Supernatant

The supernatant of *L. plantarum* 8014 and 14917 overnight culture was harvested and sterilized with a vacuum filter system (0.22 µm PES, Corning™ Disposable Vacuum Filter Systems, USA). *S. mutans* and *C. albicans* with a range of concentration (10^1-8^ for *S. mutans* and 10^1-6^ for *C. albicans*) were treated with the supernatant of *L. plantarum* and allowed to grow for 24 h in TSBYE with 1% glucose or 1% sucrose condition in 96-well plates. Clear culture indicated no growth of microorganisms.

### 2.5 Transcriptome Analysis by RNA-seq

#### 2.5.1 RNA Library Preparation and Sequencing

The mass of biofilms was harvested from four discs for each condition. The discs were immersed in RNAlater (Applied Biosystems/Ambion, Austin, TX, USA) for 1 h, followed by biomass removal with a spatula. RNAs were extracted and purified with MasterPure complete DNA and RNA purification kit (Epicentre, Lucigen, WI, USA). The raw RNA product was quantified using NanoDrop One Microvolume UV-Vis Spectrophotometer (Thermo Scientific™, Wilmington, DE, USA). rRNA depletion was performed using Ribo-Zero rRNA Removal Kit (Illumina, San Diego, CA, USA). The RNA sequencing library was prepared using NEBNext Ultra RNA Library Prep Kit for Illumina by following the manufacturer’s recommendations (NEB, Ipswich, MA, USA). The sequencing libraries were multiplexed and clustered on one lane of a flow cell and loaded on the Illumina HiSeq instrument according to the manufacturer’s instructions.

The RNA sequencing library was prepared using the NEBNext Ultra RNA Library Prep Kit for Illumina by following the manufacturer’s recommendations (NEB, Ipswich, MA, USA). Briefly, enriched RNAs were fragmented for 15 min at 94°C. First- and second-strand cDNAs were synthesized. The cDNA fragments were end repaired and adenylated at 3′ ends, and a universal adapter was ligated to cDNA fragments, followed by index addition and library enrichment with limited cycle PCR. Sequencing libraries were validated using the Agilent TapeStation 4200 (Agilent Technologies, Palo Alto, CA, USA) and quantified by using the Qubit 2.0 Fluorometer (Invitrogen, Carlsbad, CA) as well as by quantitative PCR (Applied Biosystems, Carlsbad, CA, USA).

The sequencing libraries were multiplexed and clustered on one lane of a flow cell and loaded on the Illumina HiSeq instrument according to the manufacturer’s instructions. The samples were sequenced using a 2x150 paired-end (PE) configuration. Image analysis and base calling were conducted using the HiSeq Control Software (HCS). Raw sequence data generated from Illumina HiSeq were converted into FASTQ files and demultiplexed using Illumina’s bcl2fastq 2.17 software. The sequence reads of all samples in the study are deposited in the NCBI Sequence Read Archive (SRA) as a study under the accession number of PRJNA809829. One mismatch was allowed for index sequence identification. After demultiplexing, sequence data were checked for overall quality and yield. The sequence reads were trimmed to remove possible adapter sequences and nucleotides with poor quality using Trimmomatic v.0.36. The STAR aligner v.2.5.2b (Dobin et al., 2013) was used to map the trimmed reads to the reference genomes. Unique gene hit counts were calculated by using feature Counts from the Subread package v.1.5.2. Only unique reads within exon regions were counted. Gene hit counts were extracted, and the gene hit count table was used for downstream differential expression analysis.

Using DESeq2, a comparison of gene expression between the groups of samples was performed. The Wald test was used to generate p-values and Log2 fold changes. *S. mutans* and *C. albicans* genes with adjusted p-values (false discovery rate (FDR) p-values) < 0.05 and absolute log2 fold changes > 2 and *L. plantarum* 14917 genes with FDR p-values < 0.05 and absolute log2 fold changes > 1 were called differentially expressed genes (DEGs) for each comparison. A gene ontology (GO) analysis was performed on the statistically significant set of genes by implementing the software GeneSCF v1.1 (Subhash and Kanduri, 2016). The GO list was used to cluster the set of genes based on their biological process and determine their statistical significance. A principal component analysis (PCA) was performed using the “plotPCA” function within the DESeq2 R package. The plot shows the samples in a 2D plane spanned by their first two principal components. The top 500 genes, selected by the highest row variance, were used to generate the plot. Volcano plots were created by VolcaNoseR (https://huygens.science.uva.nl/VolcaNoseR) (Goedhart and Luijsterburg, 2020). Kyoto Encyclopedia of Genes and Genomes pathways were generated by KEGG mapper (genome.jp) and Cytoscape software version 3.8.2.

#### 2.5.2 Real-Time Reverse Transcription Polymerase Chain Reaction

Then cDNAs were synthesized using 0.2 μg of purified RNA and the Bio-Rad iScript cDNA synthesis kit (Bio-Rad Laboratories, Inc., Hercules, CA). The resulting cDNA and negative controls were amplified by quantitative amplification conditions using Applied Biosystems™ PowerTrack™ SYBR Green Master Mix and a QuantStudio™ 3 Real-Time PCR System (Thermo Fisher Scientific, USA). Each 20-µl reaction mixture included template cDNA, 10 µM each primer, and 2× SYBR Green Mix (containing SYBR Green and Taq DNA Polymerase). Unique core genes of *S. mutans*, *C. albicans*, and *L. plantarum* were used as internal reference for comparative expression calculation: *gyrA* for *S. mutans* genes (Zeng and Burne, 2013); ACT1 for *C. albicans*, and *ropB* for *L. plantarum*.

### 2.6 Statistical Analysis

To compare the abundance of *S. mutans*, *C. albicans*, and *Lactobacillus* spp. in planktonic and biofilm conditions, the CFU values were first converted to natural log values; zero values remained to be zero. The log values were compared between each group treated with *Lactobacillus* spp. to the control group using the Mann–Whitney U test after assessing the normality of data. For other measurements, such as biomass (bacteria and EPS), number and size of microcolonies, and pH value of the biofilms at specific time points, normality tests were performed first. For normally distributed data, the comparisons between groups were tested using the t-test for two groups and one-way ANOVA for more than two groups followed by *post hoc* test. For data that were not normally distributed, Kruskal–Wallis was used to compare the outcomes of more than two groups, and the Mann–Whitney U test was used for a two-group comparison. Statistical tests were two-sided with a significant level of 5%. IBM SPSS was used for statistical analyses.

## 3 Results

### 3.1 Inhibition of *C. albicans* and *S. mutans* by *Lactobacilli* in Planktonic Condition

**Figure 1:**
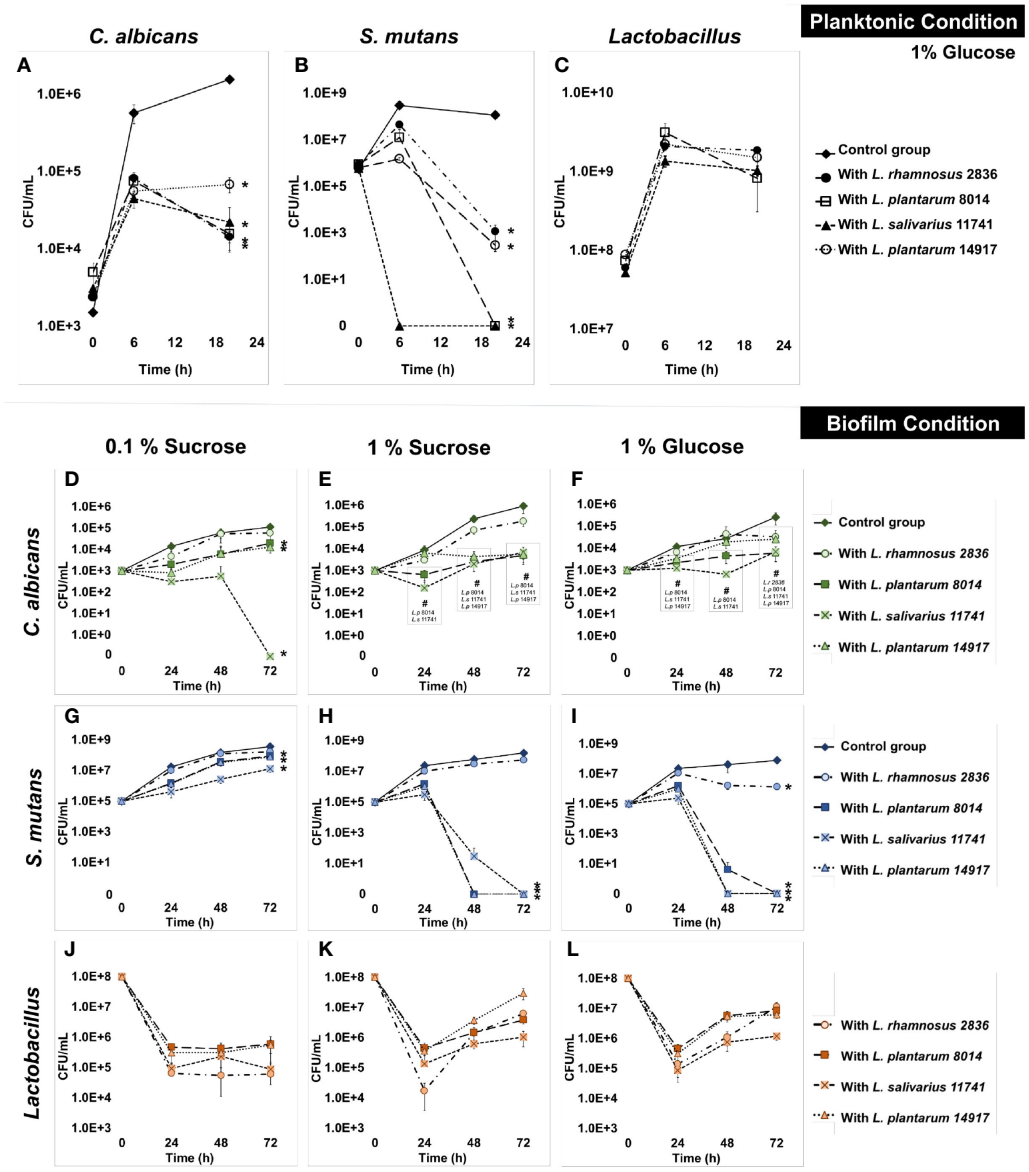
Inhibition of *C. albicans* and *S. mutans* by *Lactobacilli* in multispecies biofilms. The growth curves of *C. albicans*, *S. mutans*, and *Lactobacilli* in multispecies planktonic and biofilm conditions are plotted. The control group consists of *C. albicans* and *S. mutans*. The group with added *Lactobacilli* was marked as “with *Lactobacillus*”. **(A)** *Lactobacilli* significantly inhibited the growth of *C. a lbicans* by 1 log after 6 h and 1–2 logs after a 20-h incubation. **(B)** *Lactobacilli* significantly inhibited the growth of *S. mutans* at 6 and 20 h. *S. mutans* was inhibited to non-detectable level (<20 CFU/ml) after a 20-h incubation with *L. plantarum* 8014 and *L. salivarius* 11741. **(C)** *Lactobacilli* maintained a stable growth in all groups. **(D–L)** The growth curves of *C. albicans*, *S. mutans*, and *Lactobacilli* in multispecies biofilm conditions are plotted. **(D–F)** *Lactobacilli (L. plantarum* and *L. salivarius)* inhibited the growth of *C. albicans* in high-sucrose conditions (1%) by 72 h, a 3-log reduction compared to the control group. No difference of *C. albicans* growth was detected with the addition of *L. rhamnosus* in all sugar conditions. **(G–I)** *Lactobacilli (L. plantarum* and *L. salivarius)* inhibit the growth of *S. mutans* in high-sugar conditions (1% sucrose and 1% glucose). Significantly, *L. plantarum* 8014 and 14917 inhibited *S. mutans* in the biofilms to non-detectable level (<20 CFU/ml) as early as 48 h, and the treated biofilms remained non-detectable *S. mutans* (<20 CFU/ml) at 72 h. *L. rhamnosus* had poor performance on inhibiting the growth of *S. mutans* growth in all sugar conditions. **(J–L)** *Lactobacilli* maintained a stable growth in all groups. * Indicates that the CFU values of the multispecies biofilms were significantly less than the control group at all follow-up time points (p < 0.05). ^#^ Indicates that the CFU values of the multispecies biofilms were significantly less than the control group at specific marked time points (p < 0.05).

All four *Lactobacillus* spp. significantly inhibited the growth of *C. albicans* by 1 log at 6 h and 2 logs at 20 h (**Figure 1A**) in planktonic conditions. All tested *Lactobacilli* significantly inhibited the growth of *S. mutans* at 6 and 20 h (**Figure 1B**). In contrast to the inhibited growth of *C. albicans* and *S. mutans*, the growth of *Lactobacilli* in multispecies conditions was not different from their growth in a single *Lactobacillus* species condition (**Figure 1C**). The culture medium pH dropped faster with the addition of *Lactobacilli* spp., but reached the same acidity (~4) at 20 h across all conditions (p > 0.05, **Figure S2A**). Worth noting is that a dose-dependent effect was seen, as shown in **Figure S3**; the minimal inoculum of *L. plantarum* 14917 that demonstrated inhibition on the growth of *S. mutans* and *C. albicans* was 10^8^ CFU/ml.

### 3.2 Inhibition of *C. albicans* and *S. mutans* by *Lactobacillus* in Multispecies Biofilms

The growth of *C. albicans* and *S. mutans* was significantly inhibited by *L. salivarius* 11741, *L. plantarum* 8014, *and L. plantarum* 14917 in multispecies biofilms (**Figures 1D–L**). Interestingly, rich sucrose conditions (1% sucrose vs. 0.1% sucrose) enhanced the performance of *Lactobacillus* spp. Intriguingly, *L. plantarum* 8014 and 14917 inhibited *S. mutans* to non-detectable levels (<20 CFU/ml) as early as 48 h, and the inhibitory effect remained until 72 h, whereas *L. rhamnosus* did not inhibit the growth of *S. mutans* growth except in 1% glucose conditions. The dynamic changes in microorganism composition in each condition were plotted, as shown in **Figure S4**. In the biofilms treated with *L. salivarius* 11741*, L. plantarum* 8014, and *L. plantarum* 14917 (1% sucrose and 1% glucose conditions), *Lactobacilli* became the dominant species after 48 h. The pH of the culture medium (**Figures S2B–D**) was significantly lower with added *Lactobacilli* at 24, 48, and 72 h, compared to the control group (p < 0.05).

### 3.3 Inhibition of Cariogenic Biofilm Formation by *L. plantarum*

**Figure 2:**
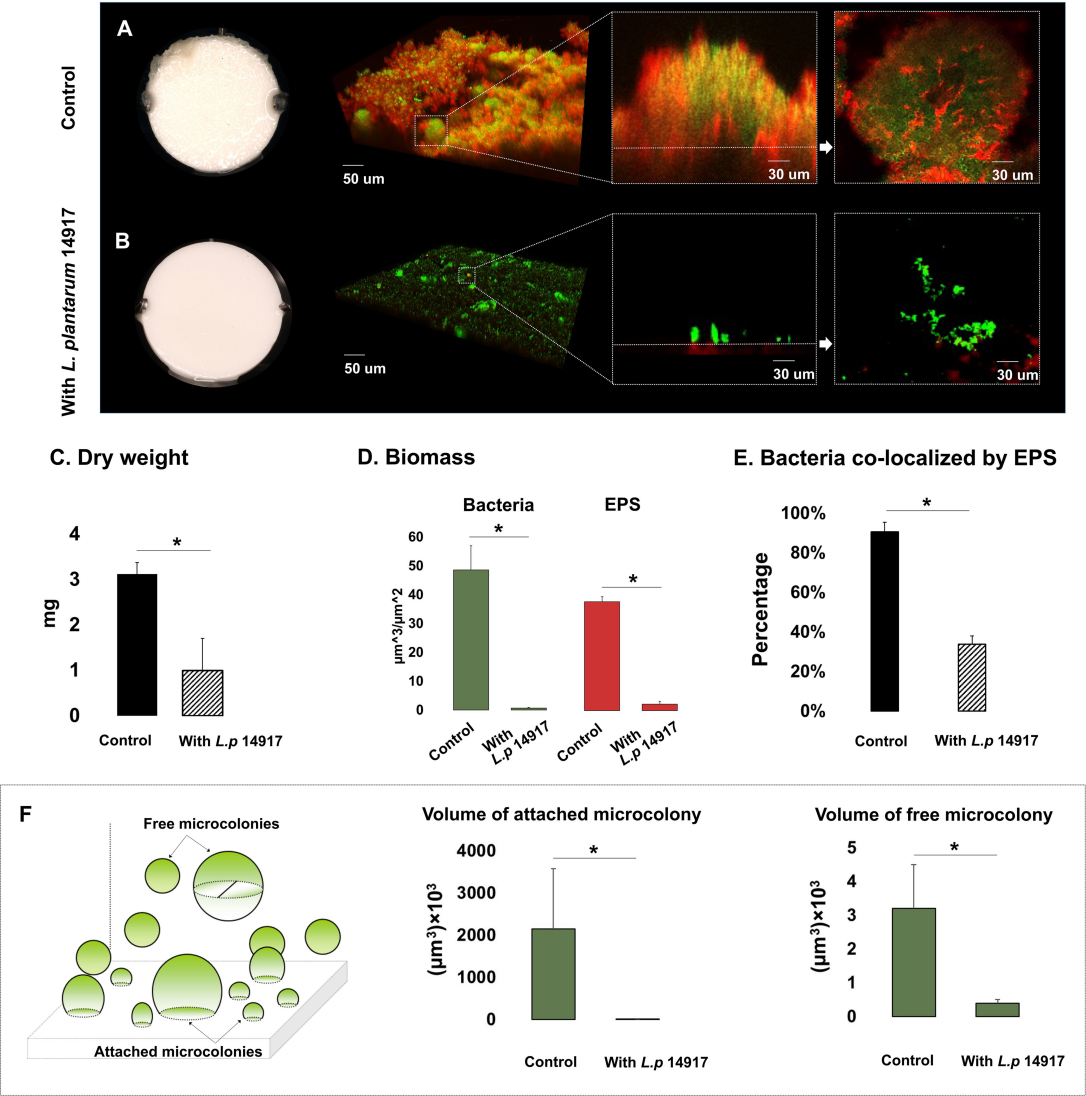
Morphogenesis, 3D architecture, and quantitative measurement of microcolonies in 72-h multispecies biofilms (1% sucrose condition). The 72-h biofilms of the control group (C*. albicans* and *S. mutans*) and experimental groups (with *L. plantarum* 14917*)* in 1% sucrose condition were visualized using a two-photon laser confocal microscope. The three-dimensional structure of the biofilms was rendered using Amira software. The green color indicates bacteria and the red color indicates the exopolysaccharides (EPS). *L. plantarum* 14917 dramatically reduced biofilm formation, compared to the control group **(A, B)**. Biofilm dry weight was significantly reduced with added *L. plantarum* 14917 **(C)**. *p < 0.05. The biomass of the two biofilm components, bacteria and exopolysaccharides (EPS), was calculated using image-processing software COMSTAT (Heydorn et al., 2000). Both *L. plantarum* 14917 significantly reduced the biomass of bacteria and EPS **(D)**. The confocal images indicate the cross-sectional and sagittal views of microcolonies formed in the control group (*S. mutans* and *C. albicans* duo-species) and with added *L. plantarum* 14917. Well-formed mushroom-shaped microcolonies were seen in the control group, and the largest size microcolonies were seen in the *S. mutans* and *C. albicans* duo-species biofilm. Microcolonies formed with added *L. plantarum* 14917 were much less structured. Bacterial components were less encapsulated with EPS. The amount of co-localization between bacteria and EPS was calculated using DUOSTAT **(E)**, which was consistent with the findings revealed in the images (*p < 0.05). The surface-attached and free-floating microcolonies were evaluated using COMSTAT and DUOSTAT software. Panel **(F)** illustrates that biofilms treated by *L. plantarum* 14917 had significantly reduced microcolony size (p > 0.05; ANOVA, comparison for all pairs using Tukey–Kramer HSD).

Since *L. plantarum* 8014 and 14917 demonstrated the better inhibition of *C. albicans* and *S. mutans* in planktonic and biofilm conditions, these two strains advanced to the biofilm structural analysis. *L. plantarum* 8014 and 14917 significantly reduced cariogenic biofilm formation measured by bacteria and EPS biomass and biofilm dry weight (p < 0.05), compared to the control group (*C. albicans–S. mutans* duo-species biofilm). The 72-h biofilms are shown in **Figure 2**, and the dynamic changes of biofilm formation from 24 to 72 h are shown in **Figure S5**. The vertical distributions of bacteria and EPS further demonstrate the altered biofilm assembly (**Figure S6**). The control group formed the thickest biofilms in 1% sucrose conditions, with the bulk of the biofilm accumulated at around 150–250 µm above the biofilm–HA disc interface (**Figures S6D, E**). Conversely, the biofilms treated by *L. plantarum* 14917 were the thinnest and had the least horizontal converge, with approximately 15% coverage of bacteria and 19% EPS at the most abundant layer (20 µm above the biofilm–HA disc interface).

Microcolonies are considered virulent and functional structures of biofilm. Surface-attached and free-floating microcolonies were identified in the biofilms. Well-formed mushroom-shaped microcolonies formed in the control group (**Figure 2A**). Microcolonies formed with added *L. plantarum* 14917 were less structured, with less bacteria components enmeshed with EPS (**Figure 2E**) (p < 0.05). Furthermore, biofilms treated with *L. plantarum* 14917 had significantly fewer surface-attached and free-floating microcolonies, with reduced size (**Figure 2F**).

### 3.4 Inhibition on *C. albicans* and *S. mutans* Growth by *L. plantarum* Supernatant

The supernatant of *L. plantarum* 14917 demonstrated antibacterial and antifungal activity against *C. albicans* and *S. mutans* (**Table S5**). Specifically, the supernatant of *L. plantarum* 14917 inhibited the growth of *S. mutans* with a starting concentration equal or lower than 10^4^ CFU/ml in 1% sucrose conditions, and the growth of *C. albicans* with a starting concentration equal or lower than 10^1^ CFU/ml in 1% sucrose conditions. The supernatant of *L. plantarum* 8014 had no inhibitory effect on *C. albicans*. The inhibitory effect was identified as bacteriostatic and fungistatic.

### 3.5 Transcriptomic Analysis

**Figure 3:**
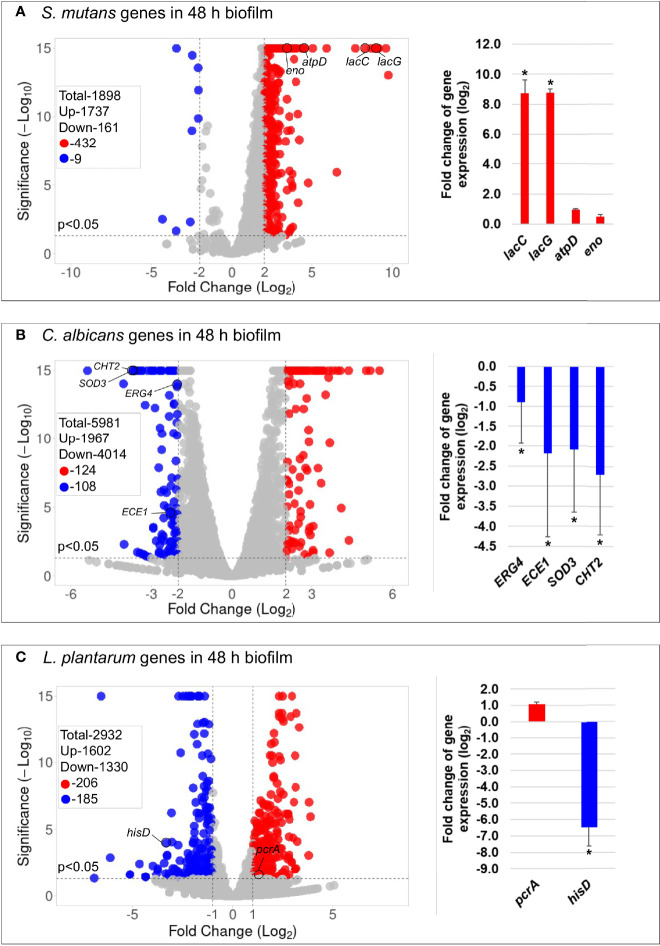
Comparison of transcriptome profiling between multispecies biofilms treated with *L. plantarum* 14917 and their controls. **(A)** Volcano plots from transcriptome analysis of *S. mutans* in multispecies (*L. plantarum* 14917 *+ S. mutans* + *C. albicans*) biofilm (48 h, 1% sucrose) compared to *S. mutans* in duo-species (*S. mutans* + *C. albicans*) biofilm. **(B)** *C. albicans* in multispecies biofilm compared to *C. albicans* in duo-species biofilm. **(C)** *L. plantarum* 14917 in multispecies biofilm compared to *L. plantarum* 14917 single-species biofilms. Data represent three independent replicates of each condition. qRT-PCR validation results of selected genes are shown on the right side of each volcano plots. * Indicates that the expression of genes in the multispecies biofilms was significantly different from that in the control group (p < 0.05).

The principal component analysis (PCA) (**Figure S8**) and the hierarchical clustering analysis (**Figure S9**) indicated distinctive transcriptomic profiles of biofilms treated with *L. plantarum* 14917. Overall, 441 genes of *S. mutans* and 232 genes of *C. albicans* had a differential expression between *L. plantarum* 14917-treated multispecies biofilm and the control group, while 391 genes of *L. plantarum* 14917 were differentially expressed between the multispecies group and *L. plantarum* 14917 single-species biofilms (**Figure 3** and **Tables S2–4**). These differentially expressed genes are defined as DEGs. The validation results from the quantitative real-time reverse transcription polymerase chain reaction (qRT-PCR) for selected genes of interest were consistent with the RNA-Seq data (**Figure 3**). Worth noting is that genes related to *C. albicans* resistance to antifungal medication (ERG4), fungal cell wall chitin remodeling (CHT2), and resistance to oxidative stress (CAT1) were significantly downregulated when treated with *L. plantarum* 14917.

**Figure 4:**
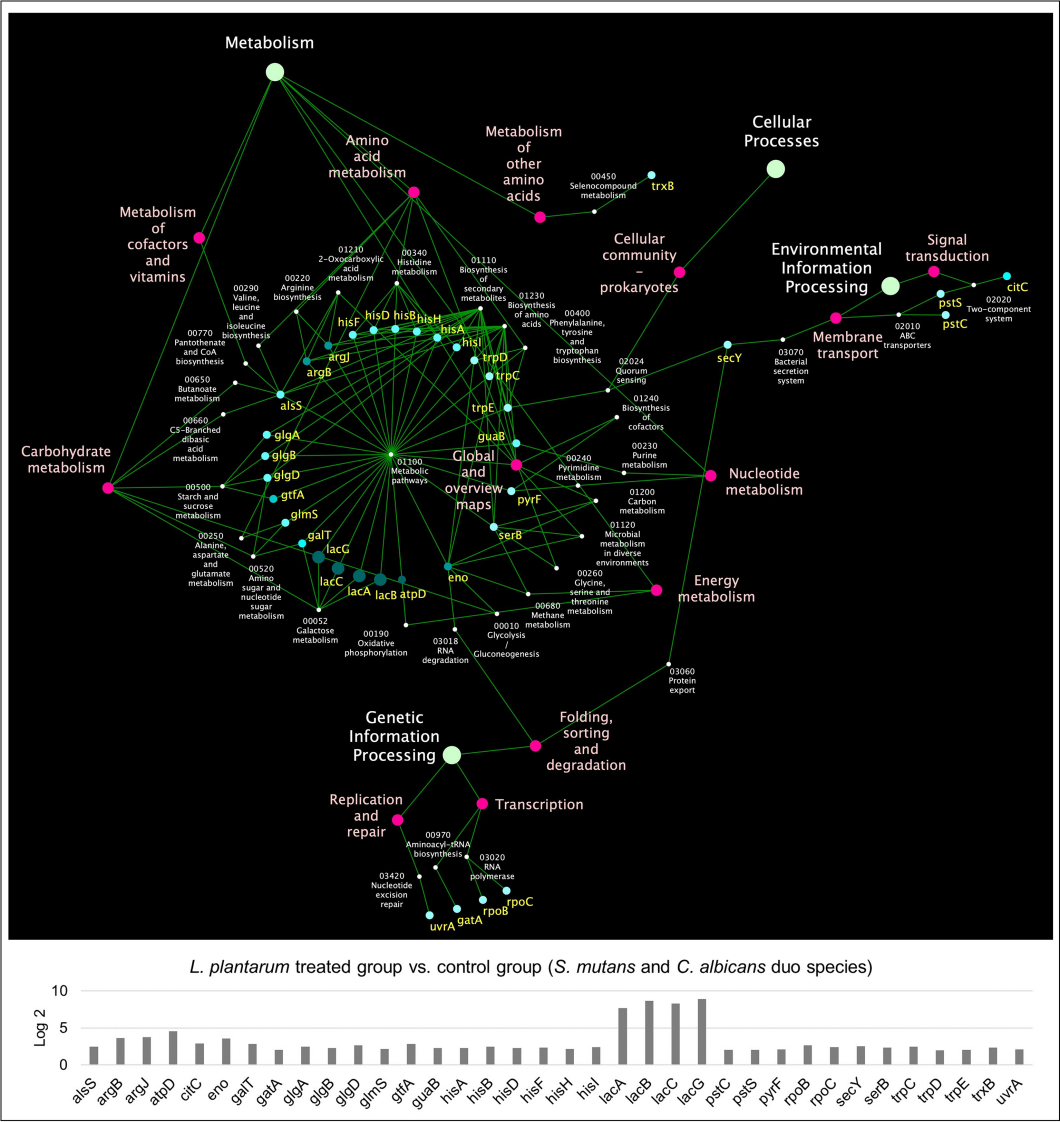
KEGG pathway network for *S. mutans* differentially expressed genes between the multispecies and duo-species biofilms. The genes of *S. mutans* differentially expressed genes between the comparison groups with FDR p-values < 0.05 and log2 fold changes > 2 were defined as DEGs and are listed in **Supplementary Table 2**. Overall, 33 impacted pathways were found for 441 *S. mutans* DEGs. The fold change of the DEGs involved in the identified pathways is shown in the lower panel.

**Figure 5:**
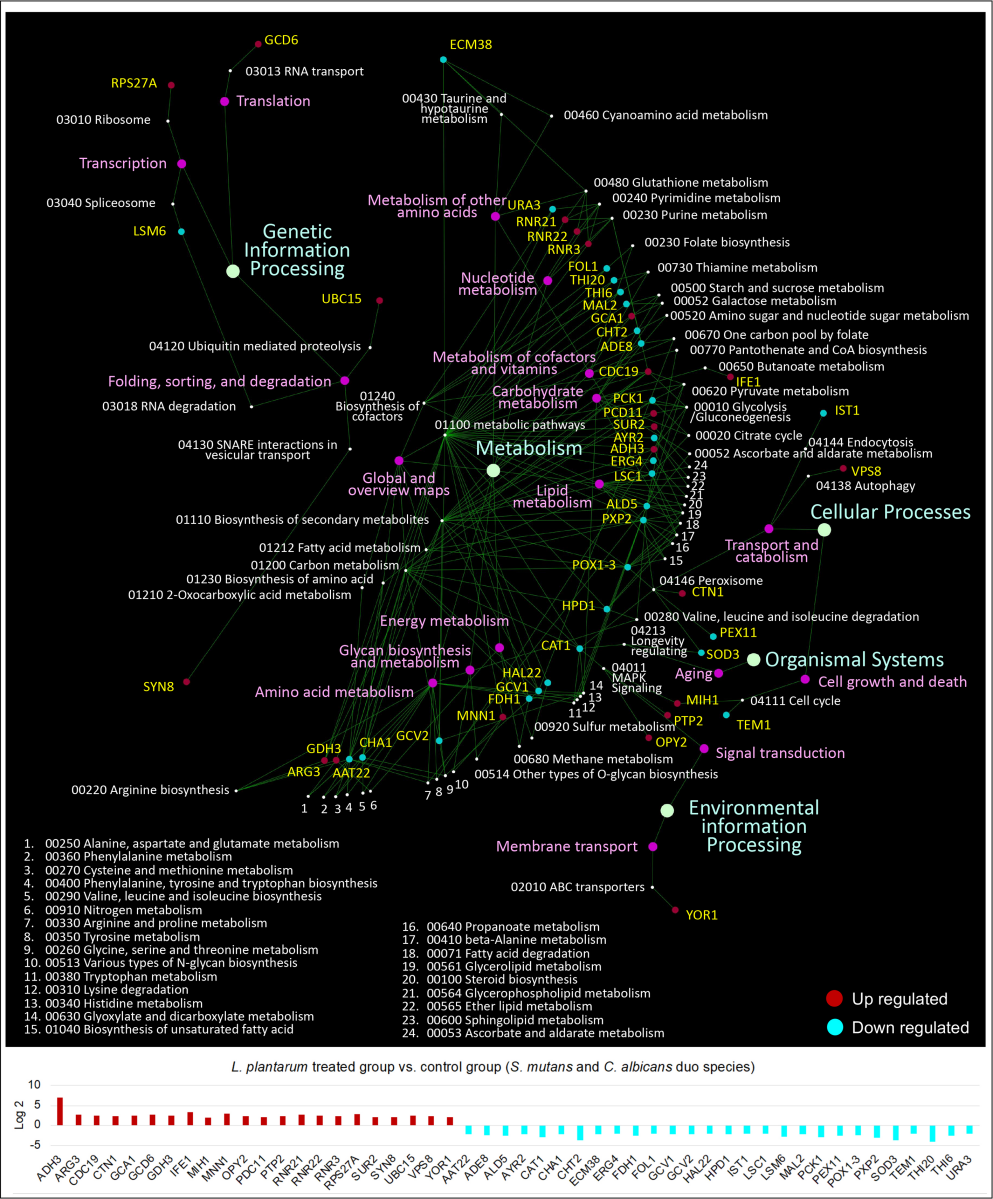
KEGG pathway network for *C. albicans* differentially expressed genes between the multispecies and duo-species biofilms. The genes of *C. albicans* that differentially expressed between the comparison groups with FDR p-values < 0.05 and log2 fold changes > 2 were defined as DEGs and are listed in **Supplementary Table 3**. Overall, 66 impacted pathways were found for 232 C*. albicans* DEGs. The fold change of the DEGs involved in the identified pathways is shown in the lower panel.

KEGG pathway analyses were further performed with 441 *S. mutans* DEGs, 232 C*. albicans* DEGs, and 391 *L. plantarum* 14917 DEGs, resulting in 33 pathways for *S. mutans*, 66 pathways for *C. albicans*, and 31 pathways for *L. plantarum* 14917. Transcriptomic analysis revealed the disruption of *S. mutans* and *C. albicans* cross-kingdom interactions with added *L. plantarum*. Genes of *S. mutans* (**Figure 4**) and *C. albicans* (**Figure 5**) involved in metabolic pathways (e.g., EPS formation, carbohydrate metabolism, glycan biosynthesis, and metabolism) were significantly downregulated. In contrast, genes of *L. plantarum* 14917 in the pathways of genetic information processing, environmental information processing, cellular processes, and metabolism (lipid, carbohydrate, glycan, energy) were significantly upregulated (**Figure S10**).

To determine the transcriptomic dynamic changes in genes of interest during specific stages of biofilm formation, particularly with the significant drop of pH value in the culture media, qRT-PCR was performed for biofilms at 50 and 52 h (2 and 4 h after culture medium change, **Figure S11**). *S. mutans* genes related to EPS formation (*gtfB* and *gtfC*) were significantly downregulated at 50 h. Genes related to *C. albicans* resistance fungal cell wall chitin remodeling (CHT2) and resistance to oxidative stress (CAT1) were also significantly downregulated following culture medium change. *Lactobacillus* genes *plnD*, *plnG*, and *plnN* that contribute to antimicrobial peptide plantaricins were significantly upregulated.

## 4 Discussion

We used a comprehensive approach to examine the inhibitory effect of probiotic *Lactobacilli* on the growth of *C. albicans* and *S. mutans* in cariogenic mixed-species biofilms. Among the four tested *Lactobacillus* spp., *L. plantarum* 14917 exhibited superior inhibitory properties, whereas *L. rhamnosus*, a commonly used probiotic in commercial products, was not capable of inhibiting the growth of *C. albicans* and *S. mutans* in cariogenic biofilms. *L. plantarum* has various potential pharmaceutical usages with recent adoption in clinical studies and trials to prevent and treat respiratory diseases, irritable bowel syndrome, depression, etc. (Arasu et al., 2016). The following mechanisms suggest its antifungal and antibiofilm activities observed in our study.

1) Production of plantaricins. Bacteriocins, antimicrobial molecules, produced by *L. plantarum* are known as plantaricins (Sabo et al., 2014). Not surprisingly, most *pln* genes (*plnD*, *plnG*, *plnN*, and *plnEF*) that encode plantaricins by *L. plantarum* were upregulated in our multispecies biofilms in comparison to *L. plantarum* 14917 single-species biofilms. The only exception is *plnA. plnA* functions as a peptide pheromone that induces transcription of *pln* genes organized in the following five operons: *plnABCD*, *plnEFI*, *plnJKLR*, *plnMNOP*, and *plnGHSTUV* (Diep et al., 2003). The possible reason for the downregulation of *plnA* still needs more exploration. Moreover, culture medium was changed at 48 h in our model. The expressions of *plnD*, *plnG*, *plnN*, *plnA*, and *plnEF* at 52 h were lower than 50 h, possibly due to the availability of culture medium sources and the pH-related expression difference.

2) Altered fitness and virulence of *S. mutans* with the addition of *L. plantarum* 14917. First, *S. mutans* upregulates specific adaptation mechanisms (e.g., F-ATPase system, fatty acid biosynthesis) to cope with acidic environments (Baker et al., 2017). Among the F-ATPase system, *atpD* has a critical function in the assembly of the ATPase complex and is highly induced at low pH (Kuhnert et al., 2004). With added *L. plantarum* 14917, *atpD* was upregulated at the 48-h biofilm but downregulated at the 50- and 52-h biofilm. Second, *S. mutans* genes that are associated with glucan synthesis and remodeling (*gtfBC*, *dexA*) and glucan-binding (*gbpB)* are usually more expressed in multispecies conditions, resulting in more abundant EPS formation in biofilms and dental plaques (Bowen and Koo, 2011). When treated with *L. plantarum* 14917, *gtfBC* of *S. mutans* were downregulated, explaining significantly reduced biofilm biomass and thickness. Third, *S. mutans lac* genes are associated with galactose metabolism and are upregulated in the presence of *C. albicans* (He et al., 2017). The addition of *L. plantarum* 14917 further enhanced the upregulation of *S. mutans lacC*G, possibly due to the relief of catabolite repression. Furthermore, studies have indicated that some probiotic lactobacilli have an ability to co-aggregate with *S. mutans* (Keller et al., 2011) and *C. albicans* (do Carmo et al., 2016), which might explain the inhibition of adhesion to the HA disc.

3) Altered *C. albicans* virulence. Significantly, several virulence genes of *C. albicans* were downregulated with added *L. plantarum* 14917: a) genes related to hyphal growth and adhesion to host cells, including the hyphal wall protein 1 gene (HWP1) and the extent of cell elongation gene 1 (ECE1) (Wang et al., 2017; Lohse et al., 2018). The inhibition of *C. albicans* switching from yeast to hyphal form was observed in planktonic conditions when treated with *L. plantarum* 14917 (**Figure S12**). b) Superoxide dismutase 3 gene (SOD3), a copper fist transcription factor; c) regulator of copper transport protein gene (CTR1); d) chitinase 2 precursor gene (CHT2) that relates to cell wall chitin remodeling (McCreath et al., 1995); and e) ergosterol biosynthesis of ERG4, a gene related to antifungal medication resistance (Copping et al., 2005).

4) Production of other antimicrobial products such as hydrogen peroxide and lactic acid. Hydrogen peroxide is a commonly suggested antimicrobial compound produced from *Lactobacilli* (Zhang et al., 2018); the inhibition of *S. mutans* in the biofilm conditions could be a result of reduced transcription of polysaccharide intercellular adhesions. Lactic acid inhibits the growth and production of virulence factors of bacteria (Zhang et al., 2018). Despite aciduric characteristics of *S. mutans* and *C. albicans*, the presence of *Lactobacilli* further decreased the pH, compared to the control group, which might affect the expression of virulence factors of *S. mutans* and *C. albicans*.

5) Sugar metabolism. The ability of *L. plantarum* to colonize different niches is associated with its ability to ferment a variety of sugars and compete against other microorganisms (Garcia-Gonzalez et al., 2021). Sucrose metabolism might be an important ecological fitness determinant of *L. plantarum* in the competitive growth in multiple-species biofilm (Yin et al., 2018). Our study revealed an interesting finding that *L. plantarum* could inhibit *S. mutans* and *C. albicans* in high-sucrose (1%) conditions but not in low-sucrose (0.1%) conditions. The pH-dependent plantaricin antimicrobial activity could be the partial reason, since plantaricin was most active at pH 5.0 (Kato et al., 1994), which is the culture medium acidity seen in 1% sucrose (pH~5), not in 0.1% sucrose (pH~6.5). In addition, *L. plantarum* exhibits remarkable genetic and phenotypic diversity, particularly in strain-specific carbohydrate utilization capacities (Fuhren et al., 2020). A previous study demonstrated that strain-specific phenotypes and strain genotypes are associated with utilization of isomaltose and other oligosaccharides (Fuhren et al., 2020). Therefore, we speculate that *L. plantarum* inhibits *S. mutans* and *C. albicans* in high-sucrose conditions due to the utilization of sugar resources and related phenotypic presentation. Future efforts should investigate the genetic traits of *L. plantarum* related to this phenomenon.

Worth noting is that our study results revealed antimicrobial properties of the overnight culture supernatant of *L. plantarum*, which supports further analysis of the supernatant in identifying active antimicrobial compounds. Furthermore, we demonstrated the dose-dependent inhibition of *L. plantarum* on the growth of *S. mutans* and *C. albicans*, where a threshold (10^8^ CFU/ml) of *L. plantarum* is needed to demonstrate the inhibitory effect in our mixed-species model that mimicked high risk for dental caries. More interestingly, an ecological shift of the microbial community was seen in our model. Despite the inhibition of *S. mutans* and *C. albicans* by a high dose of *L. plantarum* (≥10^8^ CFU/ml), a low dose of L. plantarum (104–6 CFU/ml) promoted the growth of *S. mutans* and *C. albicans* in 1% glucose planktonic conditions (**Figure S3**), which warrants further understanding of the mechanistic interaction between *L. plantarum* and other species.

Moreover, further studies are needed to assess the efficacy of inhibition of *L. plantarum* on clinical isolates of *S. mutans* and *C. albicans*. Animal studies are also warranted to test the efficacy and side effects of using probiotics in caries prevention and optimizing dosage and delivery methods. Subsequently, clinical trials combined with observation of overall oral microflora changes with probiotics will provide a deeper understanding of utilizing probiotic regimens to create and maintain oral microbial homeostasis and prevent dental caries.

## 5 Conclusions

*L. plantarum* demonstrated superior inhibition on the growth of *C. albicans* and *S. mutans*, disruption of virulent biofilm structure with reduced EPS, and virulent microcolony formation. Transcriptomic analysis further revealed disruption of *S. mutans*–*C. albicans* cross-kingdom interactions with added *L. plantarum*. Our study findings laid a critical foundation for future assessment of using *L. plantarum* 14917 as a novel caries prevention strategy in animal and clinical studies.

## Data Availability Statement

The sequence reads of all samples in the study are deposited in the NCBI Sequence Read Archive (SRA) as a study under the accession number of PRJNA809829.

## Author Contributions

YZ and JX contributed to the conception, design, data acquisition, analysis, and interpretation, drafting, and critical revision of the manuscript. TW contributed to data acquisition, analysis, and interpretation, drafting, and critical revision of the manuscript. AF, NA, ER, SQ, JB, and CG contributed to data acquisition, data interpretation, and critical review of the manuscript. All authors have read and approved the final version of the manuscript and agree to be accountable for all aspects of the work.

## Funding

JX’s research was supported by NIDCR (K23DE027412). TW’s work is supported by a grant from the National Science Foundation NSF-CCF-1934962. The funding agencies had no role in the study design, data collection, analyses, decision to publish, or preparation of the manuscript.

## Conflict of Interest

The authors declare that the research was conducted in the absence of any commercial or financial relationships that could be construed as a potential conflict of interest.

## Publisher’s Note

All claims expressed in this article are solely those of the authors and do not necessarily represent those of their affiliated organizations, or those of the publisher, the editors and the reviewers. Any product that may be evaluated in this article, or claim that may be made by its manufacturer, is not guaranteed or endorsed by the publisher.
