# Supplementary material for: Lactobacillus plantarum Disrupts S. mutans–C. albicans Cross-Kingdom Biofilms
Source: Front Cell Infect Microbiol. 2022 Mar 22;12:872012. doi: 10.3389/fcimb.2022.872012 (PMC8980721; doi:10.3389/fcimb.2022.872012)
Supplement: Supplementary file 1 [file DataSheet_1.docx]

**Title: *Lactobacillus plantarum* disrupts *S. mutans*-*C. albicans* cross-kingdom biofilms**

**Running title: Probiotics inhibit cariogenic biofilms**

**Supplemental Figures, Tables and Appendix 1 (Additional Description for Methods Section)**

**Supplemental Figures:**

**Figure S1. Schematic study design**

**A. Planktonic condition.** *Candida albicans*, *Streptococcus mutans* and one of the *Lactobacilli* were grown in 1% glucose planktonic condition. **B. Biofilm condition.** The saliva-coated hydroxyapatite disc, consisting of similar components in tooth enamel, was used as the substrate for biofilm formation. *C. albicans*, *S. mutans*, and one of the *Lactobacilli* were grown in 0.1% sucrose without disturbance until 24h for initial biofilm establishment. The culture medium was changed once daily. In the sugar challenge condition, the culture medium was changed to 1% sucrose or 1% glucose at 24h and 48h. In both planktonic and biofilm conditions, the inoculation quantity of *C. albicans* (10^3^ CFU/ml) and *S. mutans* (10^5^ CFU/ml) was chosen to mimic high caries risk condition in the clinical setting. The inoculation quantity of *Lactobacillus* (10^8^ CFU/ml) is the lower range of the probiotics used in the commercial probiotic products (10^9^-10^12^ CFU/ml).

**Figure S2. pH in the culture medium of planktonic and biofilm conditions**

(A) The culture medium pH dropped faster with the addition of *Lactobacilli in* planktonic condition, however, reached the same acidic level at 20h across groups (p>0.05). (B-D) The culture medium pH of biofilms was significantly lower with added *Lactobacilli,* comparing to the control group, at 24, 48 and 72h (p<0.05).

* Indicates the pH values of the multispecies biofilms were significantly different from the pH values of the control group at all follow-up time points (p<0.05).

**Figure S3. Dose-depend effect of *Lactobacillus plantarum* 14917 on *S. mutans* and *C. albicans* in planktonic condition**

A range of inoculum of 10^4^-10^8^ CFU/ml *Lactobacillus plantarum* 14917 was used to examine the inhibitory effect on *S. mutans* and *C. albicans* grown in 1% glucose. The inhibitor effect was seen when the minimal inoculum of 10^8^ CFU/ml *L. plantarum* 14917, where pH dropped faster than other conditions.

**Figure S4. Change of species composition in multispecies biofilms**

The composition of each microorganism in different sugar conditions are plotted. In the groups with *L. salivarius* 11741*, L. plantarum* 8014 and 14917 in both 1% sucrose and 1% glucose conditions, *Lactobacilli* became the dominate species after 48 h’ incubation (E-F, H-I, K-L).

**Figure S5. Dynamics of the morphogenesis and 3D architecture development of multispecies biofilms**

The biofilms of the control group (C*. albicans* and *S. mutans*) and experimental groups (with *L. plantarum* 8014 and 14917*)* were visualized by two-photon laser confocal microscope. The three-dimensional structure of the biofilms was rendered using Amira software. The green color indicates bacteria and the red color indicates the exopolysaccharides (EPS). *L. plantarum* 8014 and 14917 dramatically reduced biofilm formation at 48h and 72h in 1% sucrose condition, comparing to the control group (E and F). Biofilm dry weight was significantly reduced with added *L. plantarum* 8014 and 14917 (D). * p<0.05. The biomass of the two biofilm components, bacteria and exopolysaccharides (EPS), was calculated using image-processing software COMSTAT (Heydorn *et al.*, 2000). Both *L. plantarum* 8014 and 14917 significantly reduced the biomass of bacteria (C) and EPS (D) at 48h and 72h in 1% sucrose condition. The average thickness of the biofilms was reduced with added *L. plantarum* 8014 and 14917 (G and H). * p<0.05.

**Figure S6. Layer distribution of multispecies biofilms**

The biovolume of bacteria and EPS was measured using the image-processing software COMSTAT (Heydorn *et al.*, 2000). The vertical biofilm distribution was plotted. The control group *(S. mutans* and *C. albicans* duo-species biofilm) formed the thickest biofilms in 1% sucrose condition; the bulk of biofilms was accumulated at around 150 - 250 um above the biofilm-HA disc interface (D and E). The biofilms treated by *L. plantarum* 14917 in 1% sucrose condition (O) were the thinnest at 72h and had the least horizontal converge, with only approximate 15% coverage of bacteria and 19% EPS at the most abundant layer (20 um above the biofilm-HA disc interface).

**Figure S7. Q-Score distribution of RNA samples from 48h biofilm**

**
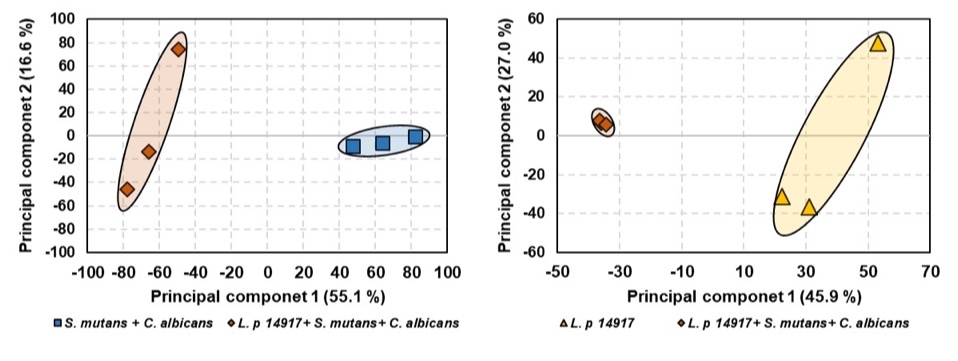
**

**Figure S8. Principal Component Analysis (PCA) for RNA samples from 48h biofilm**

**Figure S9. Hierarchical clustering analysis for RNAseq samples from 48h biofilm**

Based on gene expression in RNA-seq, hierarchical clustering of features presented the similarity in expression profiles of the features over groups. (A) hierarchical clustering of *S. mutans* and *C. albicans* genes in the control group (*S. mutans* + *C. albicans*) and treatment group (*S. mutans* + *C. albicans* + *L. plantarum* 14917). (B) hierarchical clustering of *L. plantarum* genes in the *L. plantarum* single species biofilm and treatment group (*S. mutans* + *C. albicans* + *L. plantarum* 14917).

**Figure S10. KEGG pathway network for *L. plantarum* 14917 differentially expressed genes between the multi-species and *L. plantarum* 14917 single-species biofilms**

The genes of *L. plantarum* 14917 that differentially expressed between the comparison groups with FDR p-values < 0.05 and log2 fold changes > 1 were defined as DEGs and listed in Supplementary Table 4. 31 impacted pathways were found for 391 *L. plantarum* 14917 DEGs. The fold change of the DEGs involved in the identified pathways are shown in the lower panel

**Figure S11. Transcriptomic dynamic changes of genes of interest at 50h and 52h biofilm by qRT-PCR**

To determine the transcriptomic dynamic changes in genes of interest during specific stages of biofilms formation, qRT-PCR were performed for biofilms at 50h and 52h. We compared the *S. mutans* and *C. albicans* gene expression of the multi-species biofilm (*L. plantarum 14917+S. mutans* + *C. albicans*) with the duo-species biofilm (*S. mutans* + *C. albicans*), as well as *L. plantarum* 14917 gene expression of the multi-species biofilm (*L. plantarum* 14917*+S. mutans* + *C. albicans*) with the *L. plantarum* 14917 single species biofilm.

* Indicates that expression of genes in the multi-species biofilms was significantly different from that in the control group (p < 0.05).

**Figure S12. Inhibition of *C. albicans* hyphae formation with added *L. plantarum* 14917**

(A) *S. mutans* and *C. albicans* grown in 1% glucose at 20h. (B) *S. mutans* and *C. albicans* grown in 1% glucose with added *L. plantarum* 14917 at 20h. The addition of *L. plantarum* 14917 reduced the growth of *C. albicans* and inhibited the switching from yeast to hyphae form.

**Appendix 1: Additional Description for Methods Section**

**Laser scanning confocal fluorescence microscopy (LCSFM) imaging of biofilm matrix**

We assessed two essential components of biofilm matrix: bacteria and exopolysaccharides (EPS) using LCSFM, methods detailed previously (Xiao et al., 2012) and in the appendix. Briefly, 1 μM Alexa Fluor^®^ 647-labeled dextran conjugate (Molecular Probes, Invitrogen Corp., Carlsbad, CA) was added to the culture medium from the beginning of and during the development of the biofilms for exopolysaccharides visualization. The bacterial species and fungal species were labeled by SYTO^®^ 9 green fluorescent nucleic acid stain (485/498nm; Molecular Probes). The images were obtained using an Olympus FV 1000 two photon laser scanning microscope (Olympus, Tokyo, Japan) equipped with a 10X (0.45 numerical aperture) water immersion objective lens. Each biofilm formed on the HA disc was scanned at 5 positions randomly (Xiao and Koo, 2010). Three independent biofilm experiments were performed, and 10 image stacks were collected for each experiment. Amira 5.0.2 (Mercury Computer Systems Inc., Chelmsford, MS) was used to create 3D renderings of EPS and bacteria of the biofilms detailed previously (Xiao and Koo, 2010; Klein et al., 2011). COMSTAT and DUOSTAT (<http://www.imageanalysis.dk>) were used for biofilm quantitative analysis, including biomass, number and size (volume, diameter, and height) of microcolonies, and the co-localization of EPS and bacteria across the biofilms (Xiao et al., 2012).

**Microbiological analysis of the mixed-species bacterial population**

The biofilms were homogenized by sonication detailed previously(Xiao et al., 2012). The homogenized suspension was used to determine the number of viable cells by plating on blood agar using an automated EddyJet Spiral Plater (IUL, SA, Barcelona, Spain). Three species were differentiated by colony morphology in conjunction with microscopic examination of cells from selected colonies(Guggenheim et al., 2001).

**References:**

Guggenheim, B., Giertsen, E., Schupbach, P., and Shapiro, S. (2001). Validation of an in vitro biofilm model of supragingival plaque. *J Dent Res* 80(1)**,** 363-370.

Klein, M.I., Xiao, J., Heydorn, A., and Koo, H. (2011). An analytical tool-box for comprehensive biochemical, structural and transcriptome evaluation of oral biofilms mediated by mutans streptococci. *J Vis Exp* (47). doi: 10.3791/2512.

Xiao, J., Klein, M.I., Falsetta, M.L., Lu, B., Delahunty, C.M., Yates, J.R., 3rd, et al. (2012). The exopolysaccharide matrix modulates the interaction between 3D architecture and virulence of a mixed-species oral biofilm. *PLoS Pathog* 8(4)**,** e1002623. doi: 10.1371/journal.ppat.1002623.

Xiao, J., and Koo, H. (2010). Structural organization and dynamics of exopolysaccharide matrix and microcolonies formation by Streptococcus mutans in biofilms. *J Appl Microbiol* 108(6)**,** 2103-2113. doi: 10.1111/j.1365-2672.2009.04616.x.

**Supplemental Tables**

| **Table S1. Primers used in RT-qPCR** | | | | |
| --- | --- | --- | --- | --- |
| Genes | Primers | Sequence | Amplicon size (bp) | Source or References |
| gyrA | Sm_gyrA_F | CCAAGAATCTGCTGTCCG | 111 | [a] |
|  | Sm_gyrA_R | TTGCGACTATCTGCTATGTG |  |  |
| gtfB | Sm_gtfB_F | AGCAATGCAGCCATCTACAAAT | 96 | [b] |
|  | Sm_gtfB_R | ACGAACTTTGCCGTTATTGTCA |  |  |
| gtfC | Sm_gtfC_F | CTCAACCAACCGCCACTGTT | 91 | [b] |
|  | Sm_gtfC_R | GGTTTAACGTCAAAATTAGCTGTATTAGC |  |  |
| gtfD | Sm_gtfD_F | CACAGGCAAAAGCTGAATTAACA | 81 | [b] |
|  | Sm_gtfD_R | GAATGGCCGCTAAGTCAACAG |  |  |
| atpD | Sm_atpD_F | TGTTGATGGTCTGGGTGAAA | 176 | [c] |
|  | Sm_atpD_R | TTTGACGGTCTCCGATAACC |  |  |
| eno | Sm_eno_F | CAGCGTCTTCAGTTCCATCA | 194 | [c] |
|  | Sm_eno_R | TCACTCAGATGCTCCAATCG |  |  |
| lacG | Sm_lacG_F | ATTGGATGCGTGCTTTTGATGG | 94 | [d] |
|  | Sm_lacG_R | CGACCGACACCCTTAATCTGG |  |  |
| lacC | Sm_lacC_F | GCTGGAATTACATCGGCTCTTGC | 157 | [d] |
|  | Sm_lacC_R | CCTCCGCTACCTCAATTTGTTGG |  |  |
|  |  |  |  |  |
| ACT1 | Ca_ACT1_F | TGCTCCAGAAGAACACCCA | 182 | [e] |
|  | Ca_ACT1_R | CACCTGAATCCAAAACAATACCAGT |  |  |
| HWP1 | Ca_HWP1_F | TGGTGCTATTACTATTCCGG | 182 | [f] |
|  | Ca_HWP1_R | CAATAATAGCAGCACCGAAG |  |  |
| ECE1 | Ca_ECE1_F | GCTGGTATCATTGCTGATAT | 168 | [f] |
|  | Ca_ECE1_R | TTCGATGGATTGTTGAACAC |  |  |
| CHT2 | Ca_CHT2_F | TTGGGATGCTTCTGGGGCTT | 111 | This study |
|  | Ca_CHT2_R | GCAGAAGAAGATGGGGCAACAC |  |  |
| ERG4 | Ca_ERG4_F | TCAAATGTGCCAATGGTTCT | 101 | [g] |
|  | Ca_ERG4_R | AGCCCAAGTCAATGTTTGAA |  |  |
| SOD3 | Ca_SOD3_F | CAGTATGGGTCTGTTTCAAACCTTA | 211 | [h] |
|  | Ca_SOD3_R | GATATTGCAAGTAGTACGCATGTTC |  |  |
|  |  |  |  |  |
| rpoB | Lp14_rpoB_F | CACCGTACCCGTAGAAGTTATGC | 106 | [i] |
|  | Lp14_rpoB_R | GGAGACCTTGATCCAAGAACCA |  |  |
| hisD | Lp14_hisD_F | TGAACCACTCGGTGACTACG | 150 | [j] |
|  | Lp14_hisD_R | GGAGCTTCCTTAGCCAAAGC |  |  |
| pcrA | Lp14_pcrA_F | AGGAGGTCTGGGTCTCAACG | 118 | [i] |
|  | Lp14_pcrA_R | AAGGTCCGTTGCTCGCTAGT |  |  |
| plnD | Lp14_plnD_F | TGAGGACAAACAGACTGGAC | 415 | [k] |
|  | Lp14_plnD_R | GCATCGGAAAAATTGCGGATAC |  |  |
| plnEF | Lp14_plnEF_F | GGCATAGTTAAAATTCCCCCC | 428 | [l] |
|  | Lp14_plnEF_R | CAGGTTGCCGCAAAAAAAG |  |  |
| plnG | Lp14_plnG_F | TGCGGTTATCAGTATGTCAAAG | 454 | [l] |
|  | Lp14_plnG_R | CCTCGAAACAATTTCCCCC |  |  |
| plnN | Lp14_plnN_F | ATTGCCGGGTTAGGTATCG | 146 | [l] |
|  | Lp14_plnN_R | CCTAAACCATGCCATGCAC |  |  |
| plnA | Lp14_plnA_F | GTGGAAAGAGTAGTGCGTATTC | 135 | This study |
|  | Lp14_plnA_R | CGCCATCTATACGAAATATAACTTG |  |  |

| References: | |
| --- | --- |
| [a] | He J, Kim D, Zhou X, Ahn SJ, Burne RA, Richards VP et al (2017). RNA-Seq Reveals Enhanced Sugar Metabolism in Streptococcus mutans Co-cultured with Candida albicans within Mixed-Species Biofilms. Front Microbiol 8: 1036. |
| [b] | Ahn SJ, Lemos JAC, Burne RA (2005). Role of HtrA in growth and competence of Streptococcus mutans UA159. Journal of Bacteriology 187: 3028-3038. |
| [c] | Xu X, Zhou XD, Wu CD (2011). The Tea Catechin Epigallocatechin Gallate Suppresses Cariogenic Virulence Factors of Streptococcus mutans. Antimicrobial Agents and Chemotherapy 55: 1229-1236. |
| [d] | Zeng L, Das S, Burne RA (2010). Utilization of Lactose and Galactose by Streptococcus mutans: Transport, Toxicity, and Carbon Catabolite Repression. Journal of Bacteriology 192: 2434-2444. |
| [e] | Branco J, Martins-Cruz C, Rodrigues L, Silva RM, Araujo-Gomes N, Goncalves T et al (2021). The transcription factor Ndt80 is a repressor of Candida parapsilosis virulence attributes. Virulence 12: 601-614. |
| [f] | Wang S, Wang QY, Yang EC, Yan L, Li T, Zhuang H (2017). Antimicrobial Compounds Produced by Vaginal Lactobacillus crispatus Are Able to Strongly Inhibit Candida albicans Growth, Hyphal Formation and Regulate Virulence-related Gene Expressions. Front Microbiol 8: 11. |
| [g] | Dorsaz S, Snaka T, Favre-Godal Q, Maudens P, Boulens N, Furrer P et al (2017). Identification and Mode of Action of a Plant Natural Product Targeting Human Fungal Pathogens. Antimicrobial Agents and Chemotherapy 61. |
| [h] | Li CX, Gleason JE, Zhang SX, Bruno VM, Cormack BP, Culotta VC (2015). Candida albicans adapts to host copper during infection by swapping metal cofactors for superoxide dismutase. Proceedings of the National Academy of Sciences of the United States of America 112: E5336-E5342. |
| [i] | Marco ML, Bongers RS, de Vos WM, Kleerebezem M (2007). Spatial and temporal expression of Lactobacillus plantarum genes in the gastrointestinal tracts of mice. Appl Environ Microbiol 73: 124-132. |
| [j] | Seme H, Gjuracic K, Kos B, Fujs S, Stempelj M, Petkovic H et al (2015). Acid resistance and response to pH-induced stress in two Lactobacillus plantarum strains with probiotic potential. Beneficial Microbes 6: 369-379. |
| [k] | Ben Omar N, Abriouel H, Lucas R, Martínez-Cañamero M, Guyot J-P, Gálvez A (2006). Isolation of bacteriocinogenic Lactobacillus plantarum strains from ben saalga, a traditional fermented gruel from Burkina Faso. Int J Food Microbiol 112:44–50 |
| [l] | Paramithiotis S, Papadelli M, Pardali E, Mataragas M, Drosinos EH (2019). Evaluation of Plantaricin Genes Expression During Fermentation of Raphanus sativus Roots with a Plantaricin-Producing Lactobacillus plantarum Starter. Current Microbiology 76: 909-916. |

**Table S2. Differential gene expression of *S. mutans* grown in treatment group vs. control group.
Significant genes ( >(-)1 Log2 fold change and FDR p value<0.05) that fit KEGG pathways are shown below.**

Control group: *S. mutans*+*C. albicans*
Treatment group: *L. plantarum 14917*+*S. mutants*+*C. albicans*

| **GeneID** | **Gene name** | **Log2 fold change** | **FDR p value** | **Description** | | | |  |  |  |  |  |
| --- | --- | --- | --- | --- | --- | --- | --- | --- | --- | --- | --- | --- |
| SMU_1382 | leuC | 1.01681677 | 0.001326873 | putative 3-isopropylmalate dehydratase, large subunit | | | |  |  |  |  |  |
| SMU_48 | purD | 1.017714538 | 0.005846681 | phosphoribosyl glycinamide synthetase (GARS) | | | |  |  |  |  |  |
| SMU_1673 | upp | 1.033824554 | 0.017433909 | uracil phosphoribosyltransferase | | |  |  |  |  |  |  |
| SMU_1691 | dltA | 1.036509432 | 8.18065E-05 | putative D-alanine-D-alanyl carrier protein ligase | | | |  |  |  |  |  |
| SMU_969 | folP | 1.070306659 | 0.006358378 | dihydropteroate synthase | |  |  |  |  |  |  |  |
| SMU_364 | glnA | 1.087562779 | 0.000413263 | glutamate--ammonia ligase | |  |  |  |  |  |  |  |
| SMU_822 | rpoD | 1.130654528 | 0.000191035 | major sigma factor (sigma 70/42) | | |  |  |  |  |  |  |
| SMU_1838 | secA | 1.140850395 | 2.06053E-07 | preprotein translocase subunit SecA | | |  |  |  |  |  |  |
| SMU_25 | recO | 1.201405193 | 0.001470343 | putative DNA repair protein RecO | | |  |  |  |  |  |  |
| SMU_1656 | serC | 1.21480267 | 5.18388E-05 | putative phosphoserine aminotransferase | | | |  |  |  |  |  |
| SMU_2074 | nrdD | 1.231621429 | 2.66959E-05 | putative anaerobic ribonucleoside-triphosphate reductase | | | | |  |  |  |  |
| SMU_1234 | rpiA | 1.235584913 | 0.002169069 | putative ribose 5-phosphate isomerase A | | | |  |  |  |  |  |
| SMU_2085 | recA | 1.25144568 | 0.000105056 | recombination protein RecA | |  |  |  |  |  |  |  |
| SMU_784 | aroA | 1.282537926 | 0.004218393 | 5-enolpyruvylshikimate-3-phosphate synthase | | | |  |  |  |  |  |
| SMU_1833 | recG | 1.305276709 | 4.37609E-05 | putative ATP-dependent DNA helicase, RecG | | | |  |  |  |  |  |
| SMU_366 | gltB | 1.323663992 | 9.10561E-07 | NADPH-dependent glutamate synthase (small subunit) | | | | |  |  |  |  |
| SMU_2151 | pgsA | 1.339759291 | 4.24725E-05 | putative phosphotidylglycerophosphate synthase | | | |  |  |  |  |  |
| SMU_1123 | deoC | 1.355104185 | 1.72009E-05 | putative deoxyribose-phosphate aldolase | | | |  |  |  |  |  |
| SMU_1688 | dltD | 1.359422205 | 1.35309E-05 | putative extramembranal protein, DltD protein | | | |  |  |  |  |  |
| SMU_1737 | fabZ | 1.371713926 | 0.001534146 | putative 3-hydroxymyristoyl-(acyl carrier protein) dehydratase | | | | | |  |  |  |
| SMU_1581 | dnaX | 1.377620011 | 1.44775E-08 | DNA polymerase III, gamma/tau subunit | | |  |  |  |  |  |  |
| SMU_1839 | manA | 1.385277693 | 1.30536E-07 | mannose-6-phosphate isomerase | | |  |  |  |  |  |  |
| SMU_780 | aroC | 1.406876627 | 6.95558E-07 | putative chorismate synthase | |  |  |  |  |  |  |  |
| SMU_1066 | guaA | 1.410179698 | 4.40695E-05 | putative GMP synthase | |  |  |  |  |  |  |  |
| SMU_1525 | murA | 1.437187833 | 6.06969E-06 | putative UDP-N-acetylglucosamine 1-carboxyvinyltransferase | | | | | |  |  |  |
| SMU_2088 | ruvA | 1.445066587 | 0.001387998 | putative Holliday junction DNA helicase RuvA | | | |  |  |  |  |  |
| SMU_744 | ftsY | 1.466445523 | 5.36794E-08 | signal recognition particle (docking protein) | | | |  |  |  |  |  |
| SMU_291 | tkt | 1.474233693 | 2.97026E-09 | transketolase |  |  |  |  |  |  |  |  |
| SMU_1740 | fabG | 1.503789859 | 7.27672E-08 | putative 3-oxoacyl-acyl-carrier-protein reductase / 3-ketoacyl-acyl carrier protein reductase | | | | | | | |  |
| SMU_1736 | accC | 1.527043929 | 1.90846E-07 | putative acetyl-CoA carboxylase biotin carboxylase subunit | | | | |  |  |  |  |
| SMU_1532 | atpF | 1.568230454 | 2.20078E-10 | FoF1 membrane-bound proton-translocating ATPase, b subunit | | | | | |  |  |  |
| SMU_1741 | fabD | 1.570053119 | 9.2186E-07 | putative malonyl-CoA (acyl-carrier-protein) transacylase | | | | |  |  |  |  |
| SMU_50 | purE | 1.582795907 | 4.35563E-06 | putative phosphoribosylaminoimidazole carboxylase, catalytic subunit | | | | | |  |  |  |
| SMU_1227 | deoD | 1.586339415 | 6.32269E-07 | putative purine nucleoside phosphorylase | | | |  |  |  |  |  |
| SMU_1635 | glmU | 1.610293337 | 1.13979E-07 | putative UDP-N-acetylglucosamine pyrophosphorylase | | | | |  |  |  |  |
| SMU_82 | dnaK | 1.610362275 | 4.09866E-08 | heat shock protein, DnaK (HSP-70) | | |  |  |  |  |  |  |
| SMU_1739 | fabF | 1.61306116 | 1.0365E-07 | putative 3-oxoacyl-(acyl-carrier-protein) synthase | | | |  |  |  |  |  |
| SMU_842 | thiI | 1.613483121 | 7.228E-06 | putative thiamine biosynthesis protein | | |  |  |  |  |  |  |
| SMU_1102 | ascB | 1.63232027 | 2.73222E-06 | 6-phospho-beta-glucosidase | |  |  |  |  |  |  |  |
| SMU_157 | cysE | 1.643565152 | 4.78461E-05 | serine O-acetyltransferase | |  |  |  |  |  |  |  |
| SMU_1241 | uvrC | 1.648550973 | 1.23925E-08 | putative excinuclease ABC (subunit C) | | |  |  |  |  |  |  |
| SMU_856 | pyrR | 1.652519292 | 6.23137E-06 | putative pyrimidine operon regulatory protein | | | |  |  |  |  |  |
| SMU_1472 | recJ | 1.658922903 | 2.25733E-05 | putative single-strand DNA-specific exonuclease RecJ | | | | |  |  |  |  |
| SMU_1690 | dltB | 1.677312087 | 4.27961E-09 | integral membrane protein possibly involved in D-alanine export | | | | | |  |  |  |
| SMU_990 | dapA | 1.704061771 | 7.19253E-09 | putative dihydrodipicolinate synthase | | |  |  |  |  |  |  |
| SMU_1819 | gatB | 1.708855585 | 1.70309E-11 | putative glutamyl-tRNA (Gln) amidotransferase subunit B | | | | |  |  |  |  |
| SMU_537 | trpB | 1.753721787 | 0.001274045 | putative tryptophan synthase, beta subunit | | | |  |  |  |  |  |
| SMU_1174 | pcrA | 1.761124323 | 1.12555E-09 | ATP-dependent DNA helicase | |  |  |  |  |  |  |  |
| SMU_1541 | pulA | 1.770303645 | 1.9828E-09 | putative pullulanase | |  |  |  |  |  |  |  |
| SMU_971 | folK | 1.774510112 | 0.014304723 | putative 2-amino-4-hydroxy-6-hydroxymethylpteridine pyrophosphokinase | | | | | | |  |  |
| SMU_1528 | atpB | 1.791551289 | 2.50617E-10 | FoF1 membrane-bound proton-translocating ATPase, beta subunit | | | | | |  |  |  |
| SMU_1565 | malQ | 1.821515822 | 6.44147E-08 | putative 4-alpha-glucanotransferase | | |  |  |  |  |  |  |
| SMU_809 | uvrB | 1.855582583 | 1.74488E-13 | helicase subunit of the DNA excision repair complex | | | | |  |  |  |  |
| SMU_26 | plsX | 1.860867717 | 1.28898E-09 | putative fatty acid/phospholipid synthesis protein | | | |  |  |  |  |  |
| SMU_1311 | asnS | 1.883170802 | 4.91074E-09 | putative asparaginyl-tRNA synthetase | | |  |  |  |  |  |  |
| SMU_465 | nadE | 1.885023644 | 8.17549E-09 | NAD(+) synthetase (nitrogen-regulatory protein) | | | |  |  |  |  |  |
| SMU_1948 | secE | 1.892612284 | 0.022051792 | putative preprotein translocase subunit SecE | | | |  |  |  |  |  |
| SMU_233 | ilvC | 1.908242337 | 1E-15 | ketol-acid reductoisomerase | |  |  |  |  |  |  |  |
| SMU_786 | pheA | 1.920912111 | 0.000146541 | putative prephenate dehydratase | | |  |  |  |  |  |  |
| SMU_234 | ilvA | 1.956355877 | 7.05766E-09 | threonine dehydratase | |  |  |  |  |  |  |  |
| SMU_1381 | leuD | 1.990560672 | 7.68971E-05 | putative 3-isopropylmalate dehydratase, small subunit | | | | |  |  |  |  |
| SMU_534 | trpD | 2.011483004 | 4.38602E-06 | putative phosphoribosyl anthranilate transferase | | | |  |  |  |  |  |
| SMU_1138 | pstS | 2.023855043 | 1.34435E-05 | putative ABC transporter, phosphate-binding protein | | | | |  |  |  |  |
| SMU_532 | trpE | 2.035313434 | 0.001411569 | putative anthranilate synthase, alpha subunit | | | |  |  |  |  |  |
| SMU_1822 | gatA | 2.043762494 | 2.75779E-13 | putative aspartyl-tRNA synthetase | | |  |  |  |  |  |  |
| SMU_1136 | pstC | 2.055762759 | 6.14276E-06 | putative phosphate ABC transporter, permease protein | | | | |  |  |  |  |
| SMU_1851 | uvrA | 2.083376318 | 1E-15 | putative excinuclease ABC (subunit A) | | |  |  |  |  |  |  |
| SMU_1222 | pyrF | 2.115320644 | 4.30544E-07 | putative orotidine-5'-decarboxylase PyrF | | | |  |  |  |  |  |
| SMU_1187 | glmS | 2.152443147 | 8.56602E-12 | glucosamine-fructose-6-phosphate aminotransferase | | | | |  |  |  |  |
| SMU_1266 | hisH | 2.159069147 | 6.02131E-08 | putative glutamine amidotransferase HisH | | | |  |  |  |  |  |
| SMU_2157 | guaB | 2.265828034 | 1E-15 | inosine monophosphate dehydrogenase | | |  |  |  |  |  |  |
| SMU_1265 | hisA | 2.277669272 | 1.86187E-07 | putative phosphoribosyl formimino-5-aminoimidazole carboxamide ribonucleotide isomerase | | | | | | | | |
| SMU_1270 | hisD | 2.29485939 | 4.80122E-12 | putative histidinol dehydrogenase | | |  |  |  |  |  |  |
| SMU_1539 | glgB | 2.317314587 | 6.92849E-11 | putative 1,4-alpha-glucan branching enzyme | | | |  |  |  |  |  |
| SMU_1269 | serB | 2.355936402 | 3.42748E-09 | putative phosphoserine phosphatase | | |  |  |  |  |  |  |
| SMU_1264 | hisF | 2.364238866 | 1.89587E-09 | putative imidazoleglycerol-phosphate synthase, cyclase subunit | | | | | |  |  |  |
| SMU_463 | trxB | 2.368706634 | 1E-15 | putative thioredoxin reductase (NADPH) | | |  |  |  |  |  |  |
| SMU_1989 | rpoC | 2.408152492 | 1E-15 | DNA-dependent RNA polymerase, beta' subunit | | | |  |  |  |  |  |
| SMU_1263 | hisI | 2.43074973 | 0.000139163 | putative phosphoribosyl-ATP pyrophosphatase / phosphoribosyl-AMP cyclohydrolase | | | | | | | |  |
| SMU_1268 | hisB | 2.473996802 | 3.29603E-08 | putative imidazoleglycerol-phosphate dehydratase | | | | |  |  |  |  |
| SMU_1536 | glgA | 2.475798348 | 3.20598E-13 | putative starch (bacterial glycogen) synthase | | | |  |  |  |  |  |
| SMU_1452 | alsS | 2.48974432 | 1E-15 | alpha-acetolactate synthase | |  |  |  |  |  |  |  |
| SMU_535 | trpC | 2.496671141 | 0.000376788 | putative indoleglycerol phosphate synthase | | | |  |  |  |  |  |
| SMU_2006 | secY | 2.528027491 | 1E-15 | putative preprotein translocase SecY protein | | | |  |  |  |  |  |
| SMU_1537 | glgD | 2.638882978 | 2.61917E-14 | putative glycogen biosynthesis protein GlgD | | | |  |  |  |  |  |
| SMU_1990 | rpoB | 2.651908055 | 1E-15 | DNA-dependent RNA polymerase, beta subunit | | | |  |  |  |  |  |
| SMU_881 | gtfA | 2.83096927 | 1E-15 | sucrose phosphorylase, GtfA | |  |  |  |  |  |  |  |
| SMU_887 | galT | 2.867840999 | 1E-15 | galactose-1-P-uridyl transferase, GalT | | |  |  |  |  |  |  |
| SMU_1010 | citC | 2.918554013 | 0.009022709 | putative citrate lyase ligase | |  |  |  |  |  |  |  |
| SMU_1247 | eno | 3.547857445 | 1E-15 | putative enolase |  |  |  |  |  |  |  |  |
| SMU_665 | argB | 3.661193878 | 0.014237402 | putative acetylglutamate kinase | | |  |  |  |  |  |  |
| SMU_664 | argJ | 3.776981821 | 1.5777E-05 | putative ornithine acetyltransferase/N-acetylglutamate synthase | | | | | |  |  |  |
| SMU_1530 | atpD | 4.536743658 | 1E-15 | FoF1 membrane-bound proton-translocating ATPase, alpha subunit | | | | | |  |  |  |
| SMU_1496 | lacA | 7.698012461 | 1E-15 | galactose-6-phosphate isomerase, subunit LacA | | | |  |  |  |  |  |
| SMU_1494 | lacC | 8.299478252 | 1E-15 | tagatose-6-phosphate kinase | |  |  |  |  |  |  |  |
| SMU_1495 | lacB | 8.682965323 | 1E-15 | galactose-6-phosphate isomerase, subunit LacB | | | |  |  |  |  |  |
| SMU_1490 | lacG | 8.905759323 | 1E-15 | 6-phospho-beta-galactosidase | | | |  |  |  |  |  |

All FDR p value that less than 1E-15 shown as 1E-15

**Table S3. Differential gene expression of *C. albicans* grown in treatment group vs. control group.
Significant genes (>(-)1 Log2 fold change and FDR p value<0.05) that fit KEGG pathways are shown below.**

Control group: *S. mutans*+*C. albicans*
Treatment group: *L. plantarum 14917*+*S. mutants*+*C. albicans*

| **GeneID** | **Gene name** | **Log2 fold change** | **FDR p value** | **Description** |  | |  | |  |  | | |  | | |  | | |  | | |  | | |
| --- | --- | --- | --- | --- | --- | --- | --- | --- | --- | --- | --- | --- | --- | --- | --- | --- | --- | --- | --- | --- | --- | --- | --- | --- |
| CAALFM_C203370WA | THI20 | -4.037802184 | 9.16711E-15 | trifunctional hydroxymethylpyrimidine kinase/phosphomethylpyrimidine kinase/thiaminase |  | |  | |  |  | | |  | | |  | | |  | | |  | | |
| CAALFM_C700110WA | SOD3 | -3.637514951 | 1E-15 | Sod3p |  | |  | |  |  | | |  | | |  | | |  | | |  | | |
| CAALFM_C504130CA | CHT2 | -3.597341351 | 1E-15 | Cht2p |  | |  | |  |  | | |  | | |  | | |  | | |  | | |
| CAALFM_C301930WA | PXP2 | -3.144801882 | 1E-15 | Pxp2p |  | |  | |  |  | | |  | | |  | | |  | | |  | | |
| CAALFM_C503110CA | NA | -3.064220499 | 0.043827949 | cal:CAALFM_C503110CA mRNA splicing protein | | | | | | | |  | | |  | | |  | | |  | | |  |
| CAALFM_C106810WA | CAT1 | -3.006437713 | 1E-15 | catalase A | | |  | |  |  | | |  | | |  | | |  | | |  | | |
| CAALFM_C102270CA | NA | -2.977441064 | 1E-15 | hypothetical protein | |  | |  | | |  | | |  | | |  | | |  | | |  |  |
| CAALFM_CR00200WA | PCK1 | -2.93839048 | 1E-15 | phosphoenolpyruvate carboxykinase | | | |  | | |  | | |  | | |  | | |  | | |  |  |
| CAALFM_C102510WA | NA | -2.71404618 | 0.018466164 | hypothetical protein | |  | |  | | |  | | |  | | |  | | |  | | |  |  |
| CAALFM_CR00240WA | LSM6 | -2.712734391 | 0.023826076 | U4/U6-U5 snRNP complex subunit | | | |  | | |  | | |  | | |  | | |  | | |  |  |
| CAALFM_CR05170CA | FDH1 | -2.656840246 | 1E-15 | formate dehydrogenase (NAD+) | | | |  | | |  | | |  | | |  | | |  | | |  |  |
| CAALFM_C302860WA | THI6 | -2.647023658 | 7.70583E-07 | bifunctional hydroxyethylthiazole kinase/thiamine-phosphate diphosphorylase | | | | | | | | | | | | | | | | |  | | |  |
| CAALFM_C202970CA | ALD5 | -2.590996696 | 1E-15 | aldehyde dehydrogenase (NAD(P)(+)) | | | |  | | |  | | |  | | |  | | |  | | |  |  |
| CAALFM_C604310WA | PEX11 | -2.526971875 | 7.51547E-06 | Pex11p |  | |  | |  |  | | |  | | |  | | |  | | |  | | |
| CAALFM_C203090CA | ADE8 | -2.392002865 | 1.08555E-05 | phosphoribosylglycinamide formyltransferase | | | | | | | |  | | |  | | |  | | |  | | |  |
| CAALFM_C301960CA | POX1-3 | -2.355070358 | 1E-15 | acyl-CoA oxidase | | |  | |  |  | | |  | | |  | | |  | | |  | | |
| CAALFM_C603270CA | AYR2 | -2.308814509 | 3.92189E-05 | Ayr2p |  | |  | |  |  | | |  | | |  | | |  | | |  | | |
| CAALFM_C703650WA | NA | -2.298167135 | 0.003481937 | histidinol-phosphatase | |  | |  | | |  | | |  | | |  | | |  | | |  |  |
| CAALFM_CR10790WA | MAL2 | -2.272689494 | 2.35526E-05 | oligo-1,6-glucosidase IMA1 | |  | |  | | |  | | |  | | |  | | |  | | |  |  |
| CAALFM_C201270WA | CHA1 | -2.2491677 | 1E-15 | Cha1p |  | |  | |  |  | | |  | | |  | | |  | | |  | | |
| CAALFM_C401200CA | AAT22 | -2.246916183 | 7.30305E-08 | Aat22p |  | |  | |  |  | | |  | | |  | | |  | | |  | | |
| CAALFM_C403540CA | ECM38 | -2.244870527 | 0.000171255 | Ecm38p |  | |  | |  |  | | |  | | |  | | |  | | |  | | |
| CAALFM_C602890CA | HPD1 | -2.17374748 | 3.92568E-11 | Hpd1p |  | |  | |  |  | | |  | | |  | | |  | | |  | | |
| CAALFM_C108400CA | GCV2 | -2.166392296 | 1E-15 | glycine decarboxylase subunit P | | | |  | | |  | | |  | | |  | | |  | | |  |  |
| CAALFM_C602500CA | GCV1 | -2.152879407 | 2.49078E-13 | glycine decarboxylase subunit T | | | |  | | |  | | |  | | |  | | |  | | |  |  |
| CAALFM_C114410WA | NA | -2.13688693 | 9.20065E-05 | RNA-binding protein | |  | |  | | |  | | |  | | |  | | |  | | |  |  |
| CAALFM_C300540CA | IST1 | -2.125707537 | 0.023112855 | Ist1p |  | |  | |  |  | | |  | | |  | | |  | | |  | | |
| CAALFM_C601030WA | HAL22 | -2.098776564 | 5.86452E-05 | Hal22p |  | |  | |  |  | | |  | | |  | | |  | | |  | | |
| CAALFM_C403930CA | NA | -2.088647497 | 0.015999876 | cal:CAALFM_C403930CA RNA-binding signal recognition particle subunit | | | | | | | | | | | | | | | | |  | | |  |
| CAALFM_C301350CA | URA3 | -2.079998919 | 0.004691205 | orotidine-5'-phosphate decarboxylase | | | |  | | |  | | |  | | |  | | |  | | |  |  |
| CAALFM_C303470WA | NA | -2.075292265 | 1.1874E-05 | succinate-semialdehyde dehydrogenase (NAD(P)(+)) | | | | | | | | | | |  | | |  | | |  | | |  |
| CAALFM_C600540WA | NA | -2.072495397 | 0.000759871 | hypothetical protein | |  | |  | | |  | | |  | | |  | | |  | | |  |  |
| CAALFM_C502480WA | NA | -2.068819738 | 0.002093589 | cleavage polyadenylation factor subunit | | | |  | | |  | | |  | | |  | | |  | | |  |  |
| CAALFM_C500770CA | FOL1 | -2.047997849 | 5.59915E-11 | trifunctional dihydropteroate synthetase/dihydrohydroxymethylpterin pyrophosphokinase/dihydroneopterin aldolase | | | | | | | | | | | | | | | | | | | |  |
| CAALFM_C101690CA | LSC1 | -2.040345129 | 1E-15 | succinate--CoA ligase (GDP-forming) subunit alpha | | | | | | | |  | | |  | | |  | | |  | | |  |
| CAALFM_C300760WA | ERG4 | -2.032748313 | 9.16711E-15 | delta(24(24(1)))-sterol reductase | | | |  | | |  | | |  | | |  | | |  | | |  |  |
| CAALFM_C103080CA | TEM1 | -2.005642507 | 0.028464972 | Ras family GTPase | | |  | |  |  | | |  | | |  | | |  | | |  | | |
| CAALFM_C205250CA | AAT1 | -1.99404336 | 1E-15 | aspartate transaminase | |  | |  | | |  | | |  | | |  | | |  | | |  |  |
| CAALFM_C500430WA | MET14 | -1.978454087 | 0.000303449 | adenylyl-sulfate kinase | |  | |  | | |  | | |  | | |  | | |  | | |  |  |
| CAALFM_C114310WA | NA | -1.94282236 | 6.86071E-05 | deoxycytidine monophosphate deaminase | | | | | | | |  | | |  | | |  | | |  | | |  |
| CAALFM_C206720WA | GRE2 | -1.941942447 | 0.015795254 | Gre2p |  | |  | |  |  | | |  | | |  | | |  | | |  | | |
| CAALFM_CR10170CA | NA | -1.938733073 | 1.79461E-05 | asparaginase | | |  | |  |  | | |  | | |  | | |  | | |  | | |
| CAALFM_C600140CA | HPA2 | -1.92276268 | 0.005436964 | D-amino-acid N-acetyltransferase | | | |  | | |  | | |  | | |  | | |  | | |  |  |
| CAALFM_CR06760CA | LSC2 | -1.895370392 | 1E-15 | succinate--CoA ligase (GDP-forming) subunit beta | | | | | | | |  | | |  | | |  | | |  | | |  |
| CAALFM_C112780WA | RBK1 | -1.889738663 | 0.002268654 | putative ribokinase | | |  | |  |  | | |  | | |  | | |  | | |  | | |
| CAALFM_C210200WA | NA | -1.886892907 | 0.042923414 | dolichyl-diphosphooligosaccharide-protein glycotransferase | | | | | | | | | | |  | | |  | | |  | | |  |
| CAALFM_CR00150CA | POT1 | -1.876875335 | 1.34745E-11 | acetyl-CoA C-acyltransferase | |  | |  | | |  | | |  | | |  | | |  | | |  |  |
| CAALFM_C113070CA | FAD3 | -1.873351333 | 1.00351E-05 | Fad3p |  | |  | |  |  | | |  | | |  | | |  | | |  | | |
| CAALFM_CR10110WA | CHT3 | -1.866162064 | 2.42558E-08 | Cht3p |  | |  | |  |  | | |  | | |  | | |  | | |  | | |
| CAALFM_C307040CA | YSA1 | -1.861557474 | 0.000440015 | ADP-ribose diphosphatase | |  | |  | | |  | | |  | | |  | | |  | | |  |  |
| CAALFM_C401510WA | NA | -1.820125412 | 1.44497E-07 | oxidoreductase | | |  | |  |  | | |  | | |  | | |  | | |  | | |
| CAALFM_CR01930CA | BIO2 | -1.816945904 | 0.004462365 | biotin synthase | | |  | |  |  | | |  | | |  | | |  | | |  | | |
| CAALFM_C105650WA | NA | -1.798277383 | 0.004679217 | putative uridine kinase | |  | |  | | |  | | |  | | |  | | |  | | |  |  |
| CAALFM_C303720WA | GTT11 | -1.782616238 | 0.000195399 | Gtt11p |  | |  | |  |  | | |  | | |  | | |  | | |  | | |
| CAALFM_C202690WA | SER2 | -1.767744527 | 2.18173E-05 | phosphoserine phosphatase | |  | |  | | |  | | |  | | |  | | |  | | |  |  |
| CAALFM_C210070WA | NA | -1.687523007 | 0.014575372 | hypothetical protein | |  | |  | | |  | | |  | | |  | | |  | | |  |  |
| CAALFM_C304080WA | NA | -1.650180712 | 5.19726E-09 | ubiquinol--cytochrome-c reductase subunit 6 | | | | | | | |  | | |  | | |  | | |  | | |  |
| CAALFM_C504720CA | NA | -1.630215943 | 0.002108537 | GTP cyclohydrolase I | |  | |  | | |  | | |  | | |  | | |  | | |  |  |
| CAALFM_CR00540CA | MDH1 | -1.62941717 | 1.79162E-14 | malate dehydrogenase | |  | |  | | |  | | |  | | |  | | |  | | |  |  |
| CAALFM_C110750CA | NA | -1.626294035 | 0.009583415 | H(+)-transporting V0 sector ATPase subunit e | | | | | | | |  | | |  | | |  | | |  | | |  |
| CAALFM_CR01970CA | VMA4 | -1.624282482 | 1.26201E-09 | H(+)-transporting V1 sector ATPase subunit E | | | | | | | |  | | |  | | |  | | |  | | |  |
| CAALFM_C505300WA | RIB5 | -1.61591519 | 1.24507E-08 | riboflavin synthase | | |  | |  |  | | |  | | |  | | |  | | |  | | |
| CAALFM_C113630WA | CYB2 | -1.612220108 | 1E-15 | Cyb2p |  | |  | |  |  | | |  | | |  | | |  | | |  | | |
| CAALFM_C603880WA | NA | -1.598498926 | 0.005073669 | hypothetical protein | |  | |  | | |  | | |  | | |  | | |  | | |  |  |
| CAALFM_CR00510CA | ADE1 | -1.587938598 | 3.53614E-07 | phosphoribosylaminoimidazolesuccinocarboxamide synthase | | | | | | | | | | | | | |  | | |  | | |  |
| CAALFM_C110490WA | LKH1 | -1.586819023 | 1.35075E-06 | bifunctional aminopeptidase/epoxide hydrolase | | | | | | | |  | | |  | | |  | | |  | | |  |
| CAALFM_CR06150CA | ADE13 | -1.576667036 | 1.72884E-10 | adenylosuccinase | | |  | |  |  | | |  | | |  | | |  | | |  | | |
| CAALFM_C206960WA | SPE3 | -1.531230836 | 3.1844E-08 | spermidine synthase | |  | |  | | |  | | |  | | |  | | |  | | |  |  |
| CAALFM_C304390WA | CUP5 | -1.527158296 | 1.63449E-08 | H(+)-transporting V0 sector ATPase subunit c | | | | | | | |  | | |  | | |  | | |  | | |  |
| CAALFM_CR05780WA | VMA2 | -1.517052962 | 4.99053E-10 | H(+)-transporting V1 sector ATPase subunit B | | | | | | | |  | | |  | | |  | | |  | | |  |
| CAALFM_C504940WA | NA | -1.507280822 | 0.00251992 | hypothetical protein | |  | |  | | |  | | |  | | |  | | |  | | |  |  |
| CAALFM_C108010WA | DPM1 | -1.50643949 | 7.01378E-06 | dolichyl-phosphate beta-D-mannosyltransferase | | | | | | | |  | | |  | | |  | | |  | | |  |
| CAALFM_C407200CA | NA | -1.49715131 | 0.000804763 | putative lipid phosphatase | |  | |  | | |  | | |  | | |  | | |  | | |  |  |
| CAALFM_C702970WA | ECM39 | -1.495671575 | 0.000146565 | dolichyl-P-Man:Man(7)GlcNAc(2)-PP-dolichol alpha-1,6-mannosyltransferase | | | | | | | | | | | | | | | | |  | | |  |
| CAALFM_C208190WA | VMA5 | -1.495265525 | 0.001668262 | H(+)-transporting V1 sector ATPase subunit C | | | | | | | |  | | |  | | |  | | |  | | |  |
| CAALFM_CR04740CA | ADE6 | -1.487136973 | 7.53977E-09 | phosphoribosylformylglycinamidine synthase | | | | | | | |  | | |  | | |  | | |  | | |  |
| CAALFM_C303340CA | NA | -1.486856108 | 0.000254756 | hypothetical protein | |  | |  | | |  | | |  | | |  | | |  | | |  |  |
| CAALFM_C303040WA | AIP2 | -1.484482622 | 6.83778E-06 | D-lactate dehydrogenase | |  | |  | | |  | | |  | | |  | | |  | | |  |  |
| CAALFM_C306570CA | OPI3 | -1.481818651 | 0.002331185 | bifunctional phosphatidyl-N-methylethanolamine N-methyltransferase/phosphatidyl-N-dimethylethanolamine N-methyltransferase | | | | | | | | | | | | | | | | | | | |  |
| CAALFM_C406100WA | CWH41 | -1.479993949 | 1.05016E-05 | Cwh41p |  | |  | |  |  | | |  | | |  | | |  | | |  | | |
| CAALFM_CR09290WA | THI13 | -1.462542139 | 0.021879444 | 4-amino-5-hydroxymethyl-2-methylpyrimidine phosphate synthase | | | | | | | | | | | | | |  | | |  | | |  |
| CAALFM_C205890CA | IDP1 | -1.461129857 | 0.000140381 | isocitrate dehydrogenase (NADP(+)) | | | |  | | |  | | |  | | |  | | |  | | |  |  |
| CAALFM_C207590WA | VMA10 | -1.45163787 | 0.000119262 | H(+)-transporting V1 sector ATPase subunit G | | | | | | | |  | | |  | | |  | | |  | | |  |
| CAALFM_C111590WA | PLD1 | -1.45118166 | 2.06925E-08 | phospholipase D | | |  | |  |  | | |  | | |  | | |  | | |  | | |
| CAALFM_C103780CA | HMG1 | -1.445156331 | 2.1191E-09 | hydroxymethylglutaryl-CoA reductase (NADPH) | | | | | | | |  | | |  | | |  | | |  | | |  |
| CAALFM_C405640CA | NA | -1.428288079 | 0.02028288 | 1-(5-phosphoribosyl)-5- ((5-phosphoribosylamino)methylideneamino)imidazole-4-carboxamide isomerase | | | | | | | | | | | | | | | | | | | |  |
| CAALFM_C201550WA | SMP2 | -1.4282056 | 4.47722E-06 | phosphatidate phosphatase | |  | |  | | |  | | |  | | |  | | |  | | |  |  |
| CAALFM_C200400CA | NA | -1.425837769 | 7.74439E-08 | hypothetical protein | |  | |  | | |  | | |  | | |  | | |  | | |  |  |
| CAALFM_CR01620CA | MET6 | -1.414674059 | 3.90617E-12 | 5-methyltetrahydropteroyltriglutamate-homocysteine S-methyltransferase | | | | | | | | | | | | | | | | |  | | |  |
| CAALFM_C703710CA | PLC1 | -1.399618427 | 0.023810926 | phosphatidylinositol phospholipase C | | | |  | | |  | | |  | | |  | | |  | | |  |  |
| CAALFM_C100100CA | NA | -1.382932271 | 0.024810064 | cardiolipin synthase | |  | |  | | |  | | |  | | |  | | |  | | |  |  |
| CAALFM_C111240CA | CHO1 | -1.382781085 | 0.000274978 | CDP-diacylglycerol-serine O-phosphatidyltransferase | | | | | | | | | | |  | | |  | | |  | | |  |
| CAALFM_CR07010WA | MIS11 | -1.381503956 | 2.29631E-10 | trifunctional formate-tetrahydrofolate ligase/methenyltetrahydrofolate cyclohydrolase/methylenetetrahydrofolate dehydrogenase | | | | | | | | | | | | | | | | | | | |  |
| CAALFM_C306410CA | NA | -1.37145873 | 0.018670879 | dolichyl-diphosphooligosaccharide--protein glycotransferase | | | | | | | | | | | | | |  | | |  | | |  |
| CAALFM_CR05690WA | MCT1 | -1.371155796 | 0.028337896 | [acyl-carrier-protein] S-malonyltransferase | | | | | | | |  | | |  | | |  | | |  | | |  |
| CAALFM_C704210CA | NA | -1.363550912 | 6.23084E-07 | hypothetical protein | |  | |  | | |  | | |  | | |  | | |  | | |  |  |
| CAALFM_C306450WA | GLG2 | -1.35236794 | 0.010956196 | Glg2p |  | |  | |  |  | | |  | | |  | | |  | | |  | | |
| CAALFM_CR06860CA | ARO10 | -1.350208096 | 4.28624E-13 | phenylpyruvate decarboxylase | | | |  | | |  | | |  | | |  | | |  | | |  |  |
| CAALFM_CR05080WA | VMA13 | -1.341224989 | 0.00157761 | H(+)-transporting V1 sector ATPase subunit H | | | | | | | |  | | |  | | |  | | |  | | |  |
| CAALFM_C407110CA | PDA1 | -1.323498031 | 5.36155E-09 | pyruvate dehydrogenase (acetyl-transferring) subunit E1 alpha | | | | | | | | | | | | | |  | | |  | | |  |
| CAALFM_C400570CA | PCT1 | -1.318418708 | 0.001699111 | choline-phosphate cytidylyltransferase | | | |  | | |  | | |  | | |  | | |  | | |  |  |
| CAALFM_C306020WA | MNN9 | -1.306953633 | 0.000581425 | mannosyltransferase complex subunit | | | |  | | |  | | |  | | |  | | |  | | |  |  |
| CAALFM_C209640WA | PMI1 | -1.299460773 | 0.001418299 | mannose-6-phosphate isomerase | | | |  | | |  | | |  | | |  | | |  | | |  |  |
| CAALFM_CR09160CA | ERG13 | -1.297084143 | 4.25918E-07 | hydroxymethylglutaryl-CoA synthase | | | |  | | |  | | |  | | |  | | |  | | |  |  |
| CAALFM_C103730CA | MNS1 | -1.29643795 | 0.000788626 | mannosyl-oligosaccharide 1,2-alpha-mannosidase | | | | | | | |  | | |  | | |  | | |  | | |  |
| CAALFM_C504570CA | URA7 | -1.296395969 | 1.91556E-08 | CTP synthase | | |  | |  |  | | |  | | |  | | |  | | |  | | |
| CAALFM_C100410CA | NA | -1.293151272 | 0.000113097 | hexadecenal dehydrogenase | |  | |  | | |  | | |  | | |  | | |  | | |  |  |
| CAALFM_C208610WA | ERG9 | -1.290980999 | 0.000114356 | bifunctional farnesyl-diphosphate farnesyltransferase/squalene synthase | | | | | | | | | | | | | | | | |  | | |  |
| CAALFM_C702890CA | PMT1 | -1.287570815 | 1.11892E-07 | dolichyl-phosphate-mannose-protein mannosyltransferase | | | | | | | | | | |  | | |  | | |  | | |  |
| CAALFM_C502820CA | NA | -1.284018876 | 0.016463376 | methylthioribulose 1-phosphate dehydratase | | | | | | | |  | | |  | | |  | | |  | | |  |
| CAALFM_C210350CA | ACS1 | -1.279372581 | 1.8534E-07 | acetate--CoA ligase 1 | |  | |  | | |  | | |  | | |  | | |  | | |  |  |
| CAALFM_C206700WA | AMO2 | -1.278969968 | 0.00082892 | Amo2p |  | |  | |  |  | | |  | | |  | | |  | | |  | | |
| CAALFM_C603040CA | NA | -1.264161342 | 0.01777933 | phosphatidylinositol-3,5-bisphosphate 5-phosphatase | | | | | | | | | | |  | | |  | | |  | | |  |
| CAALFM_C307830WA | FBP1 | -1.261683669 | 1.52781E-08 | fructose 1,6-bisphosphate 1-phosphatase | | | | | | | |  | | |  | | |  | | |  | | |  |
| CAALFM_C200610CA | ATP18 | -1.261628892 | 2.34355E-06 | F1F0 ATP synthase subunit i | |  | |  | | |  | | |  | | |  | | |  | | |  |  |
| CAALFM_CR07710WA | ARO2 | -1.259262082 | 1.78991E-06 | bifunctional chorismate synthase/riboflavin reductase [NAD(P)H] | | | | | | | | | | | | | |  | | |  | | |  |
| CAALFM_C300260CA | BNA4 | -1.256562005 | 7.78441E-08 | kynurenine 3-monooxygenase | | | |  | | |  | | |  | | |  | | |  | | |  |  |
| CAALFM_C306890WA | PMT2 | -1.256291434 | 1.6542E-07 | dolichyl-phosphate-mannose-protein mannosyltransferase | | | | | | | | | | |  | | |  | | |  | | |  |
| CAALFM_C201300CA | MNN24 | -1.255760659 | 0.002007609 | Mnn24p |  | |  | |  |  | | |  | | |  | | |  | | |  | | |
| CAALFM_C406400CA | VMA8 | -1.254840668 | 0.001281563 | H(+)-transporting V1 sector ATPase subunit D | | | | | | | |  | | |  | | |  | | |  | | |  |
| CAALFM_C500040CA | NA | -1.251368746 | 0.005988886 | GPI-anchor transamidase subunit | | | |  | | |  | | |  | | |  | | |  | | |  |  |
| CAALFM_C701640WA | LAT1 | -1.244120203 | 6.23456E-07 | dihydrolipoyllysine-residue acetyltransferase | | | | | | | |  | | |  | | |  | | |  | | |  |
| CAALFM_C303790WA | MEU1 | -1.236191677 | 0.000947658 | S-methyl-5-thioadenosine phosphorylase | | | | | | | |  | | |  | | |  | | |  | | |  |
| CAALFM_C203300WA | HEM2 | -1.227827082 | 0.000168215 | porphobilinogen synthase | |  | |  | | |  | | |  | | |  | | |  | | |  |  |
| CAALFM_C201430WA | APT1 | -1.226683397 | 0.009958366 | adenine phosphoribosyltransferase | | | |  | | |  | | |  | | |  | | |  | | |  |  |
| CAALFM_C301460CA | FOX3 | -1.224334133 | 1.56599E-05 | Fox3p |  | |  | |  |  | | |  | | |  | | |  | | |  | | |
| CAALFM_C400160CA | CAR2 | -1.224333075 | 1.4101E-05 | ornithine-oxo-acid transaminase | | | |  | | |  | | |  | | |  | | |  | | |  |  |
| CAALFM_CR10600CA | NA | -1.216046514 | 0.000294555 | 4-hydroxybenzoate octaprenyltransferase | | | | | | | |  | | |  | | |  | | |  | | |  |
| CAALFM_CR02360WA | IDP2 | -1.21511431 | 3.35627E-10 | isocitrate dehydrogenase (NADP(+)) | | | |  | | |  | | |  | | |  | | |  | | |  |  |
| CAALFM_C405320WA | LYS1 | -1.214896144 | 0.000637641 | saccharopine dehydrogenase (NAD+, L-lysine-forming) | | | | | | | | | | |  | | |  | | |  | | |  |
| CAALFM_C400650WA | HIS5 | -1.213497167 | 0.017674437 | histidinol-phosphate transaminase | | | |  | | |  | | |  | | |  | | |  | | |  |  |
| CAALFM_CR01060WA | LAG1 | -1.209178692 | 0.007488211 | sphingosine N-acyltransferase | | | |  | | |  | | |  | | |  | | |  | | |  |  |
| CAALFM_C204150CA | VMA7 | -1.204832161 | 0.010216935 | H(+)-transporting V1 sector ATPase subunit F | | | | | | | |  | | |  | | |  | | |  | | |  |
| CAALFM_C307640CA | FUM12 | -1.204314685 | 4.73398E-08 | Fum12p |  | |  | |  |  | | |  | | |  | | |  | | |  | | |
| CAALFM_C109640WA | ADE12 | -1.20115805 | 7.58376E-07 | adenylosuccinate synthase | |  | |  | | |  | | |  | | |  | | |  | | |  |  |
| CAALFM_C404780WA | PMT6 | -1.197452466 | 5.28721E-05 | dolichyl-phosphate-mannose-protein mannosyltransferase | | | | | | | | | | |  | | |  | | |  | | |  |
| CAALFM_C301630WA | TFP1 | -1.19619949 | 2.15994E-06 | H(+)-transporting V1 sector ATPase subunit A | | | | | | | |  | | |  | | |  | | |  | | |  |
| CAALFM_C602740WA | NA | -1.195167919 | 3.1525E-05 | hypothetical protein | |  | |  | | |  | | |  | | |  | | |  | | |  |  |
| CAALFM_C505020CA | NA | -1.179728739 | 0.00108257 | dolichyl-diphosphooligosaccharide--protein glycotransferase | | | | | | | | | | | | | |  | | |  | | |  |
| CAALFM_C105180CA | NA | -1.176576902 | 0.021173835 | hypothetical protein | |  | |  | | |  | | |  | | |  | | |  | | |  |  |
| CAALFM_C205470WA | COQ5 | -1.174113277 | 5.20165E-05 | 2-hexaprenyl-6-methoxy-1,4-benzoquinone methyltransferase | | | | | | | | | | | | | |  | | |  | | |  |
| CAALFM_C204310WA | ERG10 | -1.173730543 | 2.65188E-06 | acetyl-CoA C-acetyltransferase | | | |  | | |  | | |  | | |  | | |  | | |  |  |
| CAALFM_C505280CA | SDH4 | -1.169405514 | 0.003689115 | Sdh4p |  | |  | |  |  | | |  | | |  | | |  | | |  | | |
| CAALFM_C401870CA | ERG8 | -1.168828221 | 0.00034533 | phosphomevalonate kinase | |  | |  | | |  | | |  | | |  | | |  | | |  |  |
| CAALFM_C105610WA | NA | -1.165847741 | 0.009573264 | glutamyl-tRNA(Gln) amidotransferase subunit | | | | | | | |  | | |  | | |  | | |  | | |  |
| CAALFM_C111170WA | WBP1 | -1.164678245 | 0.002168482 | dolichyl-diphosphooligosaccharide-protein glycotransferase | | | | | | | | | | |  | | |  | | |  | | |  |
| CAALFM_C206290CA | ATP14 | -1.163613199 | 1.09111E-05 | F1F0 ATP synthase subunit h | |  | |  | | |  | | |  | | |  | | |  | | |  |  |
| CAALFM_C102600WA | SNO1 | -1.157548828 | 0.000503691 | putative pyridoxal 5'-phosphate synthase | | | | | | | |  | | |  | | |  | | |  | | |  |
| CAALFM_CR08640CA | NA | -1.157055256 | 0.000453866 | serine C-palmitoyltransferase | |  | |  | | |  | | |  | | |  | | |  | | |  |  |
| CAALFM_C304830CA | FAS2 | -1.155934554 | 1.23002E-05 | trifunctional fatty acid synthase subunit | | | |  | | |  | | |  | | |  | | |  | | |  |  |
| CAALFM_C704040CA | NPT1 | -1.149530788 | 0.001061812 | nicotinate phosphoribosyltransferase | | | |  | | |  | | |  | | |  | | |  | | |  |  |
| CAALFM_CR07090WA | STT4 | -1.145935974 | 9.66864E-06 | 1-phosphatidylinositol 4-kinase | | | |  | | |  | | |  | | |  | | |  | | |  |  |
| CAALFM_C108980CA | ZWF1 | -1.142324779 | 0.00119855 | glucose-6-phosphate dehydrogenase | | | |  | | |  | | |  | | |  | | |  | | |  |  |
| CAALFM_C601840CA | COQ3 | -1.139323797 | 0.005910175 | hexaprenyldihydroxybenzoate methyltransferase | | | | | | | |  | | |  | | |  | | |  | | |  |
| CAALFM_C402890CA | QCR9 | -1.12965372 | 9.42439E-05 | ubiquinol--cytochrome-c reductase subunit 9 | | | | | | | |  | | |  | | |  | | |  | | |  |
| CAALFM_C501520CA | GLR1 | -1.12852671 | 0.001870699 | glutathione-disulfide reductase | | | |  | | |  | | |  | | |  | | |  | | |  |  |
| CAALFM_CR09500CA | NA | -1.1260403 | 0.031850632 | putative ATP-dependent kinase | | | |  | | |  | | |  | | |  | | |  | | |  |  |
| CAALFM_C100070WA | MVD | -1.124720874 | 2.05133E-05 | diphosphomevalonate decarboxylase | | | |  | | |  | | |  | | |  | | |  | | |  |  |
| CAALFM_C113760WA | AGM1 | -1.124226099 | 0.001779879 | phosphoacetylglucosamine mutase | | | |  | | |  | | |  | | |  | | |  | | |  |  |
| CAALFM_CR09770CA | PMT5 | -1.124147735 | 0.015748609 | putative dolichyl-phosphate-mannose--protein mannosyltransferase | | | | | | | | | | | | | |  | | |  | | |  |
| CAALFM_C604030WA | ALG2 | -1.12394096 | 0.01014653 | GDP-Man:Man(1)GlcNAc(2)-PP-dolichol alpha-1,3-mannosyltransferase | | | | | | | | | | | | | |  | | |  | | |  |
| CAALFM_C400330CA | NA | -1.123726491 | 5.38757E-05 | CAALFM_C400330CA F1F0 ATP synthase subunit e | | | | | | | |  | | |  | | |  | | |  | | |  |
| CAALFM_C301820WA | NA | -1.123394233 | 0.010819574 | hypothetical protein | |  | |  | | |  | | |  | | |  | | |  | | |  |  |
| CAALFM_C105110CA | ARO4 | -1.119935102 | 1.0686E-06 | 3-deoxy-7-phosphoheptulonate synthase | | | | | | | |  | | |  | | |  | | |  | | |  |
| CAALFM_C500900CA | URA4 | -1.115977048 | 0.000197889 | dihydroorotase | | |  | |  |  | | |  | | |  | | |  | | |  | | |
| CAALFM_CR01740WA | MCI4 | -1.105195137 | 0.001357098 | Mci4p |  | |  | |  |  | | |  | | |  | | |  | | |  | | |
| CAALFM_C405240CA | VPH1 | -1.100526986 | 3.17085E-05 | H(+)-transporting V0 sector ATPase subunit a | | | | | | | |  | | |  | | |  | | |  | | |  |
| CAALFM_CR02620CA | NA | -1.100377241 | 3.92189E-05 | hypothetical protein | |  | |  | | |  | | |  | | |  | | |  | | |  |  |
| CAALFM_C702770WA | CHS1 | -1.09889456 | 6.5755E-05 | chitin synthase | | |  | |  |  | | |  | | |  | | |  | | |  | | |
| CAALFM_CR01140CA | ERG27 | -1.097511144 | 0.006255102 | 3-keto-steroid reductase | |  | |  | | |  | | |  | | |  | | |  | | |  |  |
| CAALFM_C108410CA | SAM4 | -1.095656253 | 0.00255669 | S-adenosylmethionine-homocysteine S-methyltransferase | | | | | | | | | | |  | | |  | | |  | | |  |
| CAALFM_CR00250WA | ATP17 | -1.08989629 | 1.29238E-06 | F1F0 ATP synthase subunit f | |  | |  | | |  | | |  | | |  | | |  | | |  |  |
| CAALFM_C603760CA | SHM2 | -1.085995343 | 4.46352E-07 | glycine hydroxymethyltransferase | | | |  | | |  | | |  | | |  | | |  | | |  |  |
| CAALFM_C307340WA | GCY1 | -1.085642068 | 1.93862E-07 | glycerol 2-dehydrogenase (NADP(+)) | | | |  | | |  | | |  | | |  | | |  | | |  |  |
| CAALFM_C503790WA | GUK1 | -1.081053176 | 0.033057287 | guanylate kinase | | |  | |  |  | | |  | | |  | | |  | | |  | | |
| CAALFM_C210860CA | NA | -1.077880286 | 3.44305E-05 | uroporphyrinogen decarboxylase | | | |  | | |  | | |  | | |  | | |  | | |  |  |
| CAALFM_C207550WA | NA | -1.072602556 | 2.1606E-06 | hypothetical protein | |  | |  | | |  | | |  | | |  | | |  | | |  |  |
| CAALFM_CR08550WA | NA | -1.068210637 | 0.000618079 | fructose-2,6-bisphosphatase | |  | |  | | |  | | |  | | |  | | |  | | |  |  |
| CAALFM_C103600WA | NA | -1.067351136 | 0.008281582 | dolichyl-diphosphooligosaccharide-protein glycotransferase | | | | | | | | | | |  | | |  | | |  | | |  |
| CAALFM_C700060CA | NA | -1.065668243 | 0.005238194 | adenylate kinase | | |  | |  |  | | |  | | |  | | |  | | |  | | |
| CAALFM_C406610CA | SDH12 | -1.062251195 | 3.80058E-05 | Sdh12p |  | |  | |  |  | | |  | | |  | | |  | | |  | | |
| CAALFM_C206100WA | PMT4 | -1.058803351 | 6.92684E-06 | dolichyl-phosphate-mannose-protein mannosyltransferase | | | | | | | | | | |  | | |  | | |  | | |  |
| CAALFM_C701900WA | NUC2 | -1.056527986 | 9.91217E-07 | Nuc2p |  | |  | |  |  | | |  | | |  | | |  | | |  | | |
| CAALFM_C504300CA | NA | -1.044314317 | 3.88265E-05 | metallodipeptidase | |  | |  | | |  | | |  | | |  | | |  | | |  |  |
| CAALFM_CR07620WA | AAT21 | -1.038088443 | 1.0846E-05 | aspartate transaminase | |  | |  | | |  | | |  | | |  | | |  | | |  |  |
| CAALFM_C202460WA | ERG7 | -1.034752031 | 0.000125187 | lanosterol synthase | |  | |  | | |  | | |  | | |  | | |  | | |  |  |
| CAALFM_C202030WA | ARO3 | -1.026591866 | 0.003417424 | 3-deoxy-7-phosphoheptulonate synthase | | | | | | | |  | | |  | | |  | | |  | | |  |
| CAALFM_C101050CA | NA | -1.023265909 | 0.011474463 | trans-2-enoyl-CoA reductase (NADPH) | | | |  | | |  | | |  | | |  | | |  | | |  |  |
| CAALFM_C404720WA | NA | -1.020488352 | 0.015912306 | methylenetetrahydrofolate dehydrogenase (NAD(+)) | | | | | | | | | | |  | | |  | | |  | | |  |
| CAALFM_C400200CA | MET15 | -1.019815733 | 3.48064E-05 | bifunctional cysteine synthase/O-acetylhomoserine aminocarboxypropyltransferase | | | | | | | | | | | | | | | | | | | |  |
| CAALFM_CR08210CA | ACO1 | -1.018385815 | 0.000601206 | aconitate hydratase | |  | |  | | |  | | |  | | |  | | |  | | |  |  |
| CAALFM_C501940WA | NA | -1.015101146 | 0.00308983 | bifunctional DRAP deaminase/tRNA pseudouridine synthase | | | | | | | | | | |  | | |  | | |  | | |  |
| CAALFM_C401370WA | ILV6 | -1.014758036 | 0.000794507 | acetolactate synthase regulatory subunit | | | | | | | |  | | |  | | |  | | |  | | |  |
| CAALFM_C704280CA | NA | -1.012288102 | 0.021965258 | hypothetical protein | |  | |  | | |  | | |  | | |  | | |  | | |  |  |
| CAALFM_C109490CA | GUA1 | -1.012251469 | 6.27586E-05 | GMP synthase (glutamine-hydrolyzing) | | | |  | | |  | | |  | | |  | | |  | | |  |  |
| CAALFM_C108080CA | NA | -1.01184637 | 3.51329E-05 | hypothetical protein | |  | |  | | |  | | |  | | |  | | |  | | |  |  |
| CAALFM_C201690WA | NA | -1.009844576 | 3.5967E-06 | hypothetical protein | |  | |  | | |  | | |  | | |  | | |  | | |  |  |
| CAALFM_C106550WA | GLT1 | -1.009373082 | 6.00439E-06 | glutamate synthase (NADH) | |  | |  | | |  | | |  | | |  | | |  | | |  |  |
| CAALFM_CR04090CA | ADE17 | -1.005190436 | 3.15217E-06 | bifunctional phosphoribosylaminoimidazolecarboxamide formyltransferase/IMP cyclohydrolase | | | | | | | | | | | | | | | | | | | |  |
| CAALFM_C206210CA | SER1 | -1.003572226 | 0.000100223 | O-phospho-L-serine:2-oxoglutarate transaminase | | | | | | | |  | | |  | | |  | | |  | | |  |
| CAALFM_C305760WA | NA | 1.002054887 | 2.54128E-05 | phosphopantothenate--cysteine ligase | | | |  | | |  | | |  | | |  | | |  | | |  |  |
| CAALFM_CR04590CA | PDX3 | 1.005393749 | 1.2762E-06 | pyridoxamine-phosphate oxidase | | | |  | | |  | | |  | | |  | | |  | | |  |  |
| CAALFM_C405560CA | ARO9 | 1.01130413 | 0.044833352 | aromatic-amino-acid:2-oxoglutarate transaminase | | | | | | | |  | | |  | | |  | | |  | | |  |
| CAALFM_C302280CA | GFA1 | 1.013859116 | 5.96779E-06 | glutamine--fructose-6-phosphate transaminase (isomerizing) | | | | | | | | | | | | | |  | | |  | | |  |
| CAALFM_C203040WA | PLC2 | 1.017460235 | 0.008446085 | Plc2p |  | |  | |  |  | | |  | | |  | | |  | | |  | | |
| CAALFM_C110550CA | GCA2 | 1.019597856 | 1.23939E-05 | Gca2p |  | |  | |  |  | | |  | | |  | | |  | | |  | | |
| CAALFM_C306870WA | TDH3 | 1.020538168 | 0.000543512 | glyceraldehyde-3-phosphate dehydrogenase (phosphorylating) | | | | | | | | | | | | | |  | | |  | | |  |
| CAALFM_C204230WA | BAT21 | 1.03927681 | 0.000412801 | branched-chain-amino-acid transaminase | | | | | | | |  | | |  | | |  | | |  | | |  |
| CAALFM_CR02820WA | PGM2 | 1.044987992 | 3.70413E-07 | phosphoglucomutase | |  | |  | | |  | | |  | | |  | | |  | | |  |  |
| CAALFM_C403380CA | DAO2 | 1.055049942 | 0.027341819 | Dao2p |  | |  | |  |  | | |  | | |  | | |  | | |  | | |
| CAALFM_CR00560WA | NTH1 | 1.058743119 | 6.61752E-06 | alpha,alpha-trehalase NTH1 | |  | |  | | |  | | |  | | |  | | |  | | |  |  |
| CAALFM_C113330CA | NA | 1.062368287 | 0.001344415 | spermine synthase | | |  | |  |  | | |  | | |  | | |  | | |  | | |
| CAALFM_C202980CA | DLD1 | 1.085901796 | 6.26479E-06 | Dld1p |  | |  | |  |  | | |  | | |  | | |  | | |  | | |
| CAALFM_C301300CA | NCE103 | 1.098266 | 0.017701796 | carbonate dehydratase | |  | |  | | |  | | |  | | |  | | |  | | |  |  |
| CAALFM_C105260CA | SDH1 | 1.104748436 | 2.76038E-06 | succinate dehydrogenase flavoprotein subunit | | | | | | | |  | | |  | | |  | | |  | | |  |
| CAALFM_C403330WA | IPK1 | 1.105551383 | 0.040106195 | inositol pentakisphosphate 2-kinase | | | |  | | |  | | |  | | |  | | |  | | |  |  |
| CAALFM_CR09670CA | NA | 1.118568032 | 1.18732E-05 | S-formylglutathione hydrolase | | | |  | | |  | | |  | | |  | | |  | | |  |  |
| CAALFM_CR07490CA | GLK4 | 1.138868557 | 1.59374E-07 | Glk4p |  | |  | |  |  | | |  | | |  | | |  | | |  | | |
| CAALFM_C102180WA | GAL7 | 1.146389818 | 4.35411E-08 | UDP-glucose:hexose-1-phosphate uridylyltransferase | | | | | | | | | | |  | | |  | | |  | | |  |
| CAALFM_C407050WA | YDC1 | 1.157174374 | 9.98154E-05 | alkaline dihydroceramidase | |  | |  | | |  | | |  | | |  | | |  | | |  |  |
| CAALFM_C208850CA | NA | 1.16244704 | 0.000102933 | hypothetical protein | |  | |  | | |  | | |  | | |  | | |  | | |  |  |
| CAALFM_C701800CA | PFK2 | 1.208556854 | 8.98143E-07 | 6-phosphofructokinase subunit beta | | | |  | | |  | | |  | | |  | | |  | | |  |  |
| CAALFM_CR02400WA | PHO112 | 1.235544864 | 0.025944199 | Pho112p |  | |  | |  |  | | |  | | |  | | |  | | |  | | |
| CAALFM_CR07150WA | GLK1 | 1.238797451 | 2.93059E-07 | glucokinase | | |  | |  |  | | |  | | |  | | |  | | |  | | |
| CAALFM_C207090CA | OLE2 | 1.240199413 | 3.38122E-06 | Ole2p |  | |  | |  |  | | |  | | |  | | |  | | |  | | |
| CAALFM_C200390CA | NA | 1.281867544 | 3.68162E-06 | cystathionine beta-lyase | |  | |  | | |  | | |  | | |  | | |  | | |  |  |
| CAALFM_C304350CA | NA | 1.303558932 | 0.005452536 | hypothetical protein | |  | |  | | |  | | |  | | |  | | |  | | |  |  |
| CAALFM_C405300WA | XKS1 | 1.306036913 | 2.42539E-11 | xylulokinase | | |  | |  |  | | |  | | |  | | |  | | |  | | |
| CAALFM_C209750WA | LEU42 | 1.319230257 | 1.58157E-11 | Leu42p |  | |  | |  |  | | |  | | |  | | |  | | |  | | |
| CAALFM_C503620WA | BTS1 | 1.332694981 | 0.000947658 | farnesyltranstransferase | |  | |  | | |  | | |  | | |  | | |  | | |  |  |
| CAALFM_C104770CA | ERG3 | 1.335924424 | 2.80582E-07 | C-5 sterol desaturase | |  | |  | | |  | | |  | | |  | | |  | | |  |  |
| CAALFM_CR02170WA | MET2 | 1.359025375 | 2.00658E-11 | homoserine O-acetyltransferase | | | |  | | |  | | |  | | |  | | |  | | |  |  |
| CAALFM_CR02180WA | PHO113 | 1.396397306 | 1.63235E-05 | acid phosphatase | | |  | |  |  | | |  | | |  | | |  | | |  | | |
| CAALFM_C203270WA | GPM1 | 1.422738419 | 9.65178E-09 | phosphoglycerate mutase | |  | |  | | |  | | |  | | |  | | |  | | |  |  |
| CAALFM_C502860CA | GRP2 | 1.447904703 | 1.3688E-10 | Grp2p |  | |  | |  |  | | |  | | |  | | |  | | |  | | |
| CAALFM_C204460WA | LYS22 | 1.457697724 | 0.005101852 | Lys22p |  | |  | |  |  | | |  | | |  | | |  | | |  | | |
| CAALFM_C601740CA | ATG15 | 1.526939064 | 4.73629E-10 | triglyceride lipase | | |  | |  |  | | |  | | |  | | |  | | |  | | |
| CAALFM_C505480WA | DES1 | 1.537131915 | 3.39074E-11 | Des1p |  | |  | |  |  | | |  | | |  | | |  | | |  | | |
| CAALFM_C504810WA | PFK1 | 1.537473667 | 1E-15 | 6-phosphofructokinase subunit alpha | | | |  | | |  | | |  | | |  | | |  | | |  |  |
| CAALFM_CR10360CA | CTM1 | 1.557697446 | 1E-15 | cytochrome c lysine N-methyltransferase | | | | | | | |  | | |  | | |  | | |  | | |  |
| CAALFM_C200480CA | PHHB | 1.569939415 | 1.44775E-08 | 4a-hydroxytetrahydrobiopterin dehydratase | | | | | | | |  | | |  | | |  | | |  | | |  |
| CAALFM_C700930WA | GPH1 | 1.590209269 | 1E-15 | glycogen phosphorylase | |  | |  | | |  | | |  | | |  | | |  | | |  |  |
| CAALFM_C702140WA | NA | 1.592481534 | 0.006409076 | hypothetical protein | |  | |  | | |  | | |  | | |  | | |  | | |  |  |
| CAALFM_CR04510WA | HXK2 | 1.595015323 | 1.88617E-09 | hexokinase 2 | | |  | |  |  | | |  | | |  | | |  | | |  | | |
| CAALFM_C201180WA | COX17 | 1.595405503 | 3.65246E-07 | copper metallochaperone | |  | |  | | |  | | |  | | |  | | |  | | |  |  |
| CAALFM_C111700CA | MRF1 | 1.601628933 | 4.50594E-12 | Mrf1p |  | |  | |  |  | | |  | | |  | | |  | | |  | | |
| CAALFM_C300320WA | RHR2 | 1.614174558 | 2.0253E-05 | glycerol-1-phosphatase | |  | |  | | |  | | |  | | |  | | |  | | |  |  |
| CAALFM_C502290WA | PDE1 | 1.678177166 | 0.002066978 | 3'\5'-cyclic-nucleotide phosphodiesterase PDE1 | | | | | | | |  | | |  | | |  | | |  | | |  |
| CAALFM_C108950WA | PFK26 | 1.701019047 | 2.82842E-13 | Pfk26p |  | |  | |  |  | | |  | | |  | | |  | | |  | | |
| CAALFM_C102150WA | GAL10 | 1.704223122 | 1E-15 | bifunctional UDP-glucose 4-epimerase/aldose 1-epimerase | | | | | | | | | | |  | | |  | | |  | | |  |
| CAALFM_C104320WA | GPM2 | 1.716073634 | 1.60786E-07 | Gpm2p |  | |  | |  |  | | |  | | |  | | |  | | |  | | |
| CAALFM_C210690WA | TPS3 | 1.735140026 | 1.46674E-13 | trehalose 6-phosphate synthase/phosphatase complex subunit | | | | | | | | | | | | | |  | | |  | | |  |
| CAALFM_CR00620CA | ARG1 | 1.736737856 | 1E-15 | argininosuccinate synthase | |  | |  | | |  | | |  | | |  | | |  | | |  |  |
| CAALFM_C402990CA | GST2 | 1.765909336 | 8.63761E-08 | Gst2p |  | |  | |  |  | | |  | | |  | | |  | | |  | | |
| CAALFM_C102170CA | GAL102 | 1.784622998 | 1.30165E-06 | Gal102p |  | |  | |  |  | | |  | | |  | | |  | | |  | | |
| CAALFM_CR01330WA | CPA2 | 1.877382374 | 4.42689E-12 | carbamoyl-phosphate synthase (glutamine-hydrolyzing) | | | | | | | | | | |  | | |  | | |  | | |  |
| CAALFM_C102130CA | GAL1 | 1.967393608 | 1E-15 | galactokinase | | |  | |  |  | | |  | | |  | | |  | | |  | | |
| CAALFM_C112080WA | LCB4 | 1.986216485 | 1.2386E-08 | sphinganine kinase | | |  | |  |  | | |  | | |  | | |  | | |  | | |
| CAALFM_C300800WA | MIH1 | 2.026609038 | 1E-15 | putative tyrosine protein phosphatase | | | |  | | |  | | |  | | |  | | |  | | |  |  |
| CAALFM_CR03140CA | SYN8 | 2.124386872 | 0.000483454 | syntaxin |  | |  | |  |  | | |  | | |  | | |  | | |  | | |
| CAALFM_C406570CA | PDC11 | 2.157435688 | 3.21653E-15 | indolepyruvate decarboxylase 1 | | | |  | | |  | | |  | | |  | | |  | | |  |  |
| CAALFM_C209990CA | YOR1 | 2.167354618 | 1E-15 | ATP-binding cassette transporter | | | |  | | |  | | |  | | |  | | |  | | |  |  |
| CAALFM_C111080WA | NA | 2.178964147 | 1E-15 | 6-phosphofructo-2-kinase | |  | |  | | |  | | |  | | |  | | |  | | |  |  |
| CAALFM_C202860WA | SUR2 | 2.200179705 | 1E-15 | sphingosine hydroxylase | |  | |  | | |  | | |  | | |  | | |  | | |  |  |
| CAALFM_C403890WA | PTP2 | 2.219005614 | 1E-15 | tyrosine protein phosphatase | |  | |  | | |  | | |  | | |  | | |  | | |  |  |
| CAALFM_C101740WA | CTN1 | 2.223330876 | 1E-15 | carnitine O-acetyltransferase | |  | |  | | |  | | |  | | |  | | |  | | |  |  |
| CAALFM_CR05570CA | RNR3 | 2.271005917 | 1.81095E-08 | Rnr3p |  | |  | |  |  | | |  | | |  | | |  | | |  | | |
| CAALFM_C300700WA | VPS8 | 2.282059534 | 1E-15 | CORVET complex membrane-binding subunit | | | | | | | |  | | |  | | |  | | |  | | |  |
| CAALFM_C108310WA | OPY2 | 2.295109241 | 1E-15 | Opy2p |  | |  | |  |  | | |  | | |  | | |  | | |  | | |
| CAALFM_C108340CA | NA | 2.297155364 | 1E-15 | hypothetical protein | |  | |  | | |  | | |  | | |  | | |  | | |  |  |
| CAALFM_C406120WA | GDH3 | 2.409119599 | 1E-15 | glutamate dehydrogenase (NADP(+)) | | | |  | | |  | | |  | | |  | | |  | | |  |  |
| CAALFM_C207570WA | RNR22 | 2.437091899 | 1E-15 | Rnr22p |  | |  | |  |  | | |  | | |  | | |  | | |  | | |
| CAALFM_C205460WA | CDC19 | 2.472190943 | 1E-15 | pyruvate kinase | | |  | |  |  | | |  | | |  | | |  | | |  | | |
| CAALFM_C210580WA | UBC15 | 2.499993787 | 0.000155171 | Ubc15p |  | |  | |  |  | | |  | | |  | | |  | | |  | | |
| CAALFM_C110290WA | GCA1 | 2.540554601 | 1E-15 | Gca1p |  | |  | |  |  | | |  | | |  | | |  | | |  | | |
| CAALFM_C402100CA | NA | 2.55414517 | 3.03605E-09 | glycosylphosphatidylinositol-alpha 1,4 mannosyltransferase I | | | | | | | | | | | | | |  | | |  | | |  |
| CAALFM_C108600CA | GCD6 | 2.596533174 | 1E-15 | translation initiation factor eIF2B catalytic subunit epsilon | | | | | | | | | | |  | | |  | | |  | | |  |
| CAALFM_C603230WA | ARG3 | 2.641435016 | 1E-15 | ornithine carbamoyltransferase | | | |  | | |  | | |  | | |  | | |  | | |  |  |
| CAALFM_C203010CA | RNR21 | 2.647024215 | 1E-15 | ribonucleotide-diphosphate reductase subunit | | | | | | | |  | | |  | | |  | | |  | | |  |
| CAALFM_C701670WA | NA | 2.678695186 | 1.69943E-09 | hypothetical protein | |  | |  | | |  | | |  | | |  | | |  | | |  |  |
| CAALFM_C700870WA | NA | 2.76446659 | 1.42147E-08 | guanine deaminase | |  | |  | | |  | | |  | | |  | | |  | | |  |  |
| CAALFM_C105510CA | RPS27A | 2.920252408 | 0.000307193 | ribosomal 40S subunit protein S27A | | | |  | | |  | | |  | | |  | | |  | | |  |  |
| CAALFM_C502630CA | MNN1 | 2.954151532 | 1E-15 | Mnn1p |  | |  | |  |  | | |  | | |  | | |  | | |  | | |
| CAALFM_C204480WA | NA | 3.203089156 | 1E-15 | hypothetical protein | |  | |  | | |  | | |  | | |  | | |  | | |  |  |
| CAALFM_C104750WA | IFE1 | 3.341077863 | 1.80664E-07 | putative dehydrogenase | |  | |  | | |  | | |  | | |  | | |  | | |  |  |
| CAALFM_CR07170WA | NA | 3.783659698 | 1E-15 | hypothetical protein | |  | |  | | |  | | |  | | |  | | |  | | |  |  |
| CAALFM_C204470WA | ADH3 | 6.988249653 | 1E-15 | Adh3p | |  | |  | | |  | | |  | | |  | | |  | | |  |  |

All FDR p value that less than 1E-15 shown as 1E-15

**Table S4. Differential gene expression of *L. p 14917* grown in treatment group vs. single species biofilm.
Significant genes (FDR p value<0.05) that fit KEGG pathways are shown below.**

Treatment group: *L. plantarum 14917*+*S. mutants*+*C. albicans*Single species biofilm: *L. plantarum 14917*

| **GeneID** | **Gene name** | **Log2 fold change** | **FDR p value** | **Description** |  |  |  |  |  |
| --- | --- | --- | --- | --- | --- | --- | --- | --- | --- |
| lp_2558 | hisB | -3.807260813 | 0.022979601 | imidazoleglycerol-phosphate dehydratase |  |  |  |  |  |
| lp_2559 | hisD | -3.335717827 | 0.000102245 | histidinol dehydrogenase |  |  |  |  |  |
| lp_2556 | hisA | -2.993938039 | 0.008047148 | phosphoribosylformimino-5-aminoimidazole carboxamideribotide isomerase | | | | | |
| lp_2553 | hisI | -2.913840591 | 0.049934114 | phosphoribosyl-AMP cyclohydrolase | |  |  |  |  |
| lp_0714 | phnC | -2.277358615 | 0.008083589 | phosphonates ABC transporter ATP-binding protein | | | |  |  |
| lp_2719 | purD | -2.171243908 | 0.001214832 | phosphoribosylamine--glycine ligase | |  |  |  |  |
| lp_1375 | metE | -1.93036019 | 5.46442E-06 | homocysteine S-methyltransferase (cobalamin-independent) | | | | |  |
| lp_2027 | dnaK | -1.762608907 | 1E-15 | chaperone, heat shock protein DnaK | |  |  |  |  |
| lp_1296 | hemH | -1.544658783 | 0.021293311 | ferrochelatase |  |  |  |  |  |
| lp_2336 | gshAB | -1.036619291 | 0.018672415 | glutathione synthetase |  |  |  |  |  |
| lp_1126 | cydB | -0.992079384 | 6.97961E-06 | cytochrome D ubiquinol oxidase subunit II | | |  |  |  |
| lp_2301 | recA | -0.597568379 | 0.02067548 | recombinase A |  |  |  |  |  |
| lp_0002 | dnaN | 0.545781395 | 0.038618331 | DNA-directed DNA polymerase III subunit beta | | |  |  |  |
| lp_2368 | atpF | 0.66492468 | 0.011215626 | H(+)-transporting two-sector ATPase, B subunit | | |  |  |  |
| lp_0001 | dnaA | 0.755852959 | 0.039868463 | chromosomal replication initiation protein DnaA | | |  |  |  |
| lp_1148 | gatA | 0.784668788 | 0.01846501 | aspartyl/glutamyl-tRNA amidotransferase subunit A | | | |  |  |
| lp_1627 | recG | 0.85309088 | 0.014759159 | ATP-dependent DNA helicase RecG | |  |  |  |  |
| lp_2019 | dltA | 0.875524388 | 0.006845039 | D-alanine--poly(phosphoribitol) ligase subunit 1 | | |  |  |  |
| lp_1145 | ligA | 0.909627957 | 0.006414703 | DNA ligase |  |  |  |  |  |
| lp_2370 | atpB | 0.913502941 | 0.001293784 | H(+)-transporting two-sector ATPase, A subunit | | |  |  |  |
| lp_2325 | thiI | 0.922812123 | 0.020204835 | thiamine biosynthesis protein ThiI, ATP pyrophosphatase | | | |  |  |
| lp_2187 | ileS | 0.927037453 | 0.000407359 | isoleucine-tRNA synthetase |  |  |  |  |  |
| lp_1022 | rpoC | 0.950130059 | 0.000648401 | DNA-directed RNA polymerase subunit beta' | | |  |  |  |
| lp_1025 | rpsL | 0.969127346 | 0.006759603 | 30S ribosomal protein S12 |  |  |  |  |  |
| lp_1149 | gatB | 1.074954704 | 8.80025E-05 | aspartyl/glutamyl-tRNA amidotransferase subunit B | | | |  |  |
| lp_0539 | mfd | 1.079251868 | 0.003401914 | transcription-repair coupling factor | |  |  |  |  |
| lp_2055 | rpsB | 1.093920416 | 2.28704E-06 | 30S ribosomal protein S2 |  |  |  |  |  |
| lp_1078 | rpsI | 1.196121544 | 0.000181695 | 30S ribosomal protein S9 |  |  |  |  |  |
| lp_1144 | pcrA | 1.213037617 | 0.038266271 | ATP-dependent DNA helicase PcrA | |  |  |  |  |
| lp_2087 | recJ | 1.21591613 | 0.004644325 | single-strand DNA-specific exonuclease RecJ | | |  |  |  |
| lp_2364 | atpD | 1.228455195 | 6.97961E-06 | H(+)-transporting two-sector ATPase, beta subunit | | | |  |  |
| lp_0757 | galU | 1.230050789 | 9.87208E-05 | UTP-glucose-1-phosphate uridylyltransferase | | |  |  |  |
| lp_1594 | rpmA | 1.249935588 | 8.43153E-05 | 50S ribosomal protein L27 |  |  |  |  |  |
| lp_0914 | guaA | 1.279666951 | 6.18622E-06 | GMP synthase |  |  |  |  |  |
| lp_1640 | rplS | 1.280365952 | 0.000177405 | 50S ribosomal protein L19 |  |  |  |  |  |
| lp_2271 | mutS | 1.30855843 | 0.006719188 | DNA mismatch repair protein MutS2 | |  |  |  |  |
| lp_1517 | rplT | 1.332712867 | 0.000216231 | 50S ribosomal protein L20 |  |  |  |  |  |
| lp_2264 | dapD | 1.333250967 | 0.003920142 | tetrahydrodipicolinate N-acetyltransferase | | |  |  |  |
| lp_0739 | secA | 1.340902154 | 0.000791816 | Preprotein translocase subunit SecA | |  |  |  |  |
| lp_1026 | rpsG | 1.34688381 | 5.96828E-07 | 30S ribosomal protein S7 |  |  |  |  |  |
| lp_1514 | thrS | 1.408046802 | 1.09569E-05 | threonyl-tRNA synthetase |  |  |  |  |  |
| lp_0511 | rho | 1.451910171 | 0.001727819 | transcription terminator factor Rho | |  |  |  |  |
| lp_2303 | pgsA | 1.482385925 | 0.00339555 | CDP-diacylglycerol--glycerol-3-phosphate 3-phosphatidyltransferase | | | | |  |
| lp_1032 | rpsJ | 1.509066845 | 0.001175529 | 30S ribosomal protein S10 |  |  |  |  |  |
| lp_2281 | yajC | 1.566818086 | 0.012954286 | preprotein translocase subunit YajC | |  |  |  |  |
| lp_2374 | upp | 1.593394141 | 8.21808E-05 | uracil phosphoribosyltransferase | |  |  |  |  |
| lp_0620 | rplA | 1.716740738 | 1.3674E-07 | 50S ribosomal protein L1 |  |  |  |  |  |
| lp_1063 | rplQ | 1.725927764 | 3.23467E-06 | 50S ribosomal protein L17 |  |  |  |  |  |
| lp_2331 | rpsD | 1.765429331 | 2.22137E-07 | 30S ribosomal protein S4 |  |  |  |  |  |
| lp_1060 | rpsM | 1.803516283 | 4.75051E-05 | 30S ribosomal protein S13 |  |  |  |  |  |
| lp_0705 | holB | 1.827295344 | 0.00028654 | DNA-directed DNA polymerase III subunit delta' | | |  |  |  |
| lp_0622 | rplL | 1.852572758 | 1.57201E-07 | 50S ribosomal protein L12/L7 | |  |  |  |  |
| lp_1044 | rpsQ | 1.882096965 | 7.03844E-05 | 30S ribosomal protein S17 |  |  |  |  |  |
| lp_0010 | ssb | 1.898026952 | 2.45228E-08 | single-strand DNA-binding protein | |  |  |  |  |
| lp_1034 | rplD | 1.907336968 | 4.10764E-12 | 50S ribosomal protein L4 |  |  |  |  |  |
| lp_1047 | rplE | 1.952822134 | 3.02646E-11 | 50S ribosomal protein L5 |  |  |  |  |  |
| lp_1056 | secY | 1.975526914 | 9.93047E-11 | preprotein translocase subunit SecY | |  |  |  |  |
| lp_1061 | rpsK | 2.020700704 | 3.49645E-06 | 30S ribosomal protein S11 |  |  |  |  |  |
| lp_1052 | rplR | 2.03882335 | 1.07306E-09 | 50S ribosomal protein L18 |  |  |  |  |  |
| lp_1636 | rpsP | 2.048270737 | 3.85391E-05 | 30S ribosomal protein S16 |  |  |  |  |  |
| lp_1045 | rplN | 2.098875788 | 3.52496E-07 | 50S ribosomal protein L14 |  |  |  |  |  |
| lp_1038 | rpsS | 2.214170392 | 1.85367E-05 | 30S ribosomal protein S19 |  |  |  |  |  |
| lp_1055 | rplO | 2.239572831 | 5.65933E-07 | 50S ribosomal protein L15 |  |  |  |  |  |
| lp_1040 | rpsC | 2.247059968 | 4.88383E-08 | 30S ribosomal protein S3 |  |  |  |  |  |
| lp_2698 | pyrF | 2.308190604 | 1E-15 | orotidine-5'-phosphate decarboxylase | |  |  |  |  |
| lp_1053 | rpsE | 2.310636283 | 3.53489E-11 | 30S ribosomal protein S5 |  |  |  |  |  |
| lp_1051 | rplF | 2.316673426 | 3.36176E-14 | 50S ribosomal protein L6 |  |  |  |  |  |
| lp_0619 | rplK | 2.368512172 | 1.49722E-08 | 50S ribosomal protein L11 |  |  |  |  |  |
| lp_1036 | rplB | 2.377195849 | 6.38253E-11 | 50S ribosomal protein L2 |  |  |  |  |  |
| lp_1039 | rplV | 2.41743417 | 0.000251963 | 50S ribosomal protein L22 |  |  |  |  |  |
| lp_1033 | rplC | 2.446679747 | 1.86764E-14 | 50S ribosomal protein L3 |  |  |  |  |  |
| lp_1043 | rpmC | 2.506132799 | 0.015748613 | 50S ribosomal protein L29 |  |  |  |  |  |
| lp_1050 | rpsH | 2.507536323 | 2.40674E-08 | 30S ribosomal protein S8 |  |  |  |  |  |
| lp_2125 | rpsO | 2.557914697 | 0.0008064 | 30S ribosomal protein S15 |  |  |  |  |  |
| lp_1046 | rplX | 2.640025764 | 6.89137E-07 | 50S ribosomal protein L24 |  |  |  |  |  |
| lp_1592 | rplU | 2.739977526 | 6.90674E-07 | 50S ribosomal protein L21 |  |  |  |  |  |
| lp_1041 | rplP | 2.762644966 | 7.00012E-07 | 50S ribosomal protein L16 |  |  |  |  |  |
| lp_1035 | rplW | 2.803938538 | 3.47833E-05 | 50S ribosomal protein L23 |  |  |  |  |  |

All FDR p value that less than 1E-15 shown as 1E-15

| **Table S5. Inhibition of *C. albicans* and *S. mutans* by *L. plantarum* supernatant** | | | | |
| --- | --- | --- | --- | --- |
|  |  |  |  |  |
| **Conditions** | ***S. mutans*** | | ***C. albicans*** | |
|  | 1% sucrose | 1% glucose | 1% sucrose | 1% glucose |
| Supernatant* of *L. plantarum* 8014 | 10^4^ CFU/ml | 10^1^ CFU/ml | No effect | No effect |
| Supernatant* *of L. plantarum* 14917 | 10^4^ CFU/ml | 10^4^ CFU/ml | 10^1^ CFU/ml | No effect |
|  |  |  |  |  |
| *Supernatant of *L. plantarum* overnight culture was harvested and sterilized. | | | | |
| *S. mutans* and *C. albicans* with a range of concentration (10^1^-10^8^ for *S. mutans*, 10^1^-10^6^ for *C. albicans*) was treated with the supernatant of *L. plantarum* and grew for 24 hours in 1% glucose and 1% sucrose condition. Clear culture indicated no growth of microorganisms and the highest inhibited concentrations of *S. mutans* and *C. albicans* are listed above. The clear culture was plated and incubated for additional 48 hours. Results revealed that the inhibitory effect of *S. mutans* and *C. albicans* are bacteriostatic and fungistatic. | | | | |
